# Integrated analysis of DNA methylation profiling and gene expression profiling identifies novel markers in lung cancer in Xuanwei, China

**DOI:** 10.1371/journal.pone.0203155

**Published:** 2018-10-04

**Authors:** Juan Wang, Yong Duan, Qing-He Meng, Rong Gong, Chong Guo, Ying Zhao, Yanliang Zhang

**Affiliations:** 1 Department of Clinical Laboratory, First Affiliated Hospital of Kunming Medical University, Kunming, China; 2 Yunnan Institute of Experimental Diagnosis, Kunming, China; 3 Yunnan Key Laboratory of Laboratory Medicine, Kunming, China; 4 Department of Laboratory Medicine, the University of Texas MD Anderson Cancer Center, Houston, Texas, United States of America; Chinese University of Hong Kong, HONG KONG

## Abstract

**Background:**

Aberrant DNA methylation occurs frequently in cancer. The aim of this study was to identify novel methylation markers in lung cancer in Xuanwei, China, through integrated genome-wide DNA methylation and gene expression studies.

**Methods:**

Differentially methylated regions (DMRs) and differentially expressed genes (DEGs) were detected on 10 paired lung cancer tissues and noncancerous lung tissues by methylated DNA immunoprecipitation combined with microarray (MeDIP-chip) and gene expression microarray analyses, respectively. Integrated analysis of DMRs and DEGs was performed to screen out candidate methylation-related genes. Both methylation and expression changes of the candidate genes were further validated and analyzed.

**Results:**

Compared with normal lung tissues, lung cancer tissues expressed a total of 6,899 DMRs, including 5,788 hypermethylated regions and 1,111 hypomethylated regions. Integrated analysis of DMRs and DEGs identified 45 tumor-specific candidate genes: 38 genes whose DMRs were hypermethylated and expression was downregulated, and 7 genes whose DMRs were hypomethylated and expression was upregulated. The methylation and expression validation results identified 4 candidate genes (*STXBP6*, *BCL6B*, *FZD10*, and *HSPB6*) that were significantly hypermethylated and downregulated in most of the tumor tissues compared with the noncancerous lung tissues.

**Conclusions:**

This integrated analysis of genome-wide DNA methylation and gene expression in lung cancer in Xuanwei revealed several genes regulated by promoter methylation that have not been described in lung cancer before. These results provide new insight into the carcinogenesis of lung cancer in Xuanwei and represent promising new diagnostic and therapeutic targets.

## Introduction

Lung cancer is the fastest-increasing cancer in China and, since 2004, the leading cause of cancer death [1]. The incidence of lung cancer is particularly high in certain regions of the country, especially Xuanwei and Gejiu. Xuanwei City (formerly known as Xuanwei County) is located in the northeast of Yunnan Province. The morbidity and mortality rates of lung cancer in Xunanwei have shown a clear upward trend since the mid-1970s and are the highest in China [2]. During the period from 2011 to 2013, lung cancer accounted for 63.03% of all cancer deaths in Xuanwei; the age-standardized mortality rates are 82.53/100,000 for males and 62.62/100,000 for females, rates that are 3 times and 6 times higher, respectively, than in other areas of China [3]. The high incidence and mortality rates of lung cancer have been strongly correlated with the domestic use of smoky coal for fuel in the region [4]. Lung cancer in Xuanwei also has other unique characteristics [1, 3, 5]: the incidence and mortality vary significantly among various regions; the incidence is particularly high in women, who are mostly nonsmokers, and is the highest for women in the entire country of China; the ratio of lung cancer mortality of males and females (1.09) is significantly lower than the national average (2.09); lung cancer incidence peaks at a younger age (41–50), more than 10 years younger than in other areas of the country; and adenocarcinoma is the major type. It is account for 60.6% in one study [6] and 53.3% [7] in another study. However, there is no large-scale data to study its prognosis and therapeutic effect. The 5-year survival rate is 15% of the 94 patients we have had. In this study, lung cancer in Xuanwei was defined as born in Xuanwei and lived in Xuanwei for more than 15 years.

DNA methylation refers to transfer of methyl groups from S-adenosyl methionine to the 5′ carbon of cytosine within cytosine–guanine (CpG) dinucleotides by DNA methyltransferase, the only known modification that targets the DNA itself. Compared to histones, which undergo a variety of post-translational modifications under different conditions, DNA methylation is relatively stable over a longer period and can be reversed by drugs, making it a very attractive target for therapy and promising as a marker for cancer onset, diagnosis, progression, treatment response, and prognosis [8,9].

DNA methylation is known to have profound effects on the regulation of gene expression. In general terms, methylation of promoter sequences of protein-coding genes results in transcriptional downregulation of the gene, and hypomethylation of previously methylated promoter regions permits transcription. DNA methylation changes have been shown to contribute to the onset and progression of various human cancers. Hypermethylation of promoters has been widely shown to contribute to silencing of tumor suppressor genes(TSG), and hypomethylation of promoters can lead to upregulation or activation of oncogenes during carcinogenesis. Many TSGs, such as *CDKN2A*, *FHIT*, *MGMT*, *RASSF1A*, *CDH1*, and *APC*, have been reported to be silenced by promoter hypermethylation in the development of breast cancer [10], lung cancer [11, 12], thymic epithelial tumors [13], colorectal cancer [14, 15], and esophageal squamous cell carcinoma [16]. Some oncogenes, however, such as *GADD45A*, are abnormally activated by hypomethylation, contributing to the occurrence of cancer, for example esophageal squamous cell carcinoma [17, 18].

Xuanwei is rich in smoky coal, which is the main fuel of the local residents; it is usually fired on unventilated fire pits. The burning of smoky coal can produce a large number of carcinogens; levels of benzo[*a*]pyrene, an indicator of carcinogenic polycyclic aromatic hydrocarbons, can reach as high as 19.3 μg/m^3^ [4]. Indoor air pollution has been reported to be the main risk factor for lung cancer [4], and air pollution is closely related to the abnormal DNA methylation of cancers [19]. It is not known, however, whether DNA methylation in lung cancer in Xuanwei is different from that in lung cancers in other geographic areas that are of different etiologies. In fact, little is known about the effects of exposure to smoky coal emissions on epigenetic alterations and the precise mechanisms underlying the regulation and maintenance of the methylome as well as their relationship with lung cancer in Xuanwei.

Our purpose in this study was to investigate DNA methylation of lung cancers occurring in this geographic area where the rates of morbidity and mortality from lung cancer are extremely high. We investigated genome-wide DNA methylation and gene expression in lung cancer by using methylated DNA immunoprecipitation combined with microarray (MeDIP-chip) analysis and gene expression microarray analysis. These technologies, along with bioinformatic analysis, allowed us to generate new information about genome-wide epigenetic regulation of gene expression in lung cancer in Xuanwei as well as new insights into the role of DNA methylation in its pathogenesis.

## Materials and methods

### Tissue samples

Fresh lung adenocarcinoma tissues and paired adjacent non-neoplastic lung tissues of 45 patients from Xuanwei were collected at the First Affiliated Hospital of Kunming Medical University, China, between August 2011 and December 2015. Noncancerous lung tissue was defined as apparently healthy lung tissue located at least 5 cm away from the cancerous tissue. All samples were assessed by an experienced pathologist to confirm the presence (>80%) or absence of cancer cells. The tissues were stabilized in RNAlater solution (Qiagen, Hilden, Germany) and then stored at -70°C. Clinicopathologic characteristics were extracted from the patients’ medical records (S1 Table). None of the patients had received preoperative treatment, such as chemotherapy or radiotherapy, and none suffered from immune system disease, chronic wasting disease, or other malignant tumors. Written informed consent was obtained from all participants. This study was approved by the Institutional Review Board for the Use of Human Subjects at Kunming Medical University.

### DNA, RNA, and protein extraction, cDNA synthesis, and DNA bisulfite modification

Genomic DNA (gDNA) and total RNA were extracted and purified from lung cancer and paired noncancerous lung tissues using, respectively, the QIAamp DNA Mini kit and RNeasy Mini kit (Qiagen, Hilden, Germany) according to the manufacturer’s instructions. Total protein was isolated from tissues by lysis of radio immunoprecipitation assay (RIPA) and protected by phenylmethylsulfonyl fluoride (PMSF) following manufacturer’s instructions (Beyotime, Shanghai, China). gDNA and total RNA were quantified by using a NanoDrop ND-1000 spectrophotometer (CapitalBio Nano Q, Beijing, China), and the RNA integrity was assessed by standard denaturing agarose gel electrophoresis. cDNA was synthesized by RNA reverse-transcription using the Transcriptor First Strand cDNA Synthesis kit (Roche, Mannheim, Germany). gDNA was bisulfite-modified and purified by using the Epitect Fast DNA Bisulfite kit (Qiagen) according to the manufacturer’s instructions, and its concentration was adjusted to 10 ng/L.

### MeDIP-chip

For genome-wide methylation detection, equal quantities of gDNA from each of the paired tumor and normal lung specimens from 10 patients were pooled. A total of 6 μg of the pooled gDNA from each tissue type was digested by *Mse*I, purified by using QIAquick PCR purification kit (Qiagen), and analyzed by agarose gel electrophoresis. *Mse*I-digested gDNA (1.25 μg) was subjected to immunoprecipitation with a mouse monoclonal anti-5-methylcytosine antibody (Diagenode, Liège, Belgium) and then was purified through Qiagen MinElute columns and amplified by using a GenomePlex Complete Whole Genome Amplification (WGA2) kit from Sigma-Aldrich (St Louis, MO, USA). The amplified DNA samples were purified with a QIAquick PCR purification kit. The NimbleGen Dual-Color DNA Labeling kit (Roche NimbleGen Systems, Inc., Madison, WI, USA) was used for labeling according to the manufacturer’s guidelines. Briefly, 1 μg DNA of each sample was incubated for 10 min at 98°C with 1 OD of Cy5-9mer primer (immunoprecipitation sample) or Cy3-9mer primer (input sample). Then, 100 pmol of deoxynucleoside triphosphates and 100 U of the Klenow fragment (New England Biolabs, Ipswich, MA, USA) were added, and the mix was incubated at 37°C for 3 h. The labeled DNA was purified by isopropanol/ethanol precipitation and hybridized to NimbleGen Human Meth 720K CpG RfSq Prom according to the manufacturer’s instructions. DOI: dx.doi.org/10.17504/protocols.io.rsxd6fn.

### Bioinformatic analysis

Signal intensity data were extracted from the scanned images of each array by NimbleScan MS 200 Microarray Scanner and the MS 200 Data Collection Software. Median-centering, quantile normalization, and linear smoothing were performed by the Bioconductor software packages Ringo, limma, and MEDME, respectively. Normalized log_2_-ratio data were created for each sample, and a sliding-window peak-finding algorithm provided by NimbleScan v2.5 was applied to determine the enriched peaks with specified parameters (sliding window width: 750 bp; miniprobes per peak: 2; *p*-value minimum cutoff: 2; maximum spacing between nearby probes within peak: 500 bp). A one-sided Kolmogorov–Smirnov test was applied to determine whether the probes were drawn from a significantly more positive distribution of intensity log_2_-ratios than those in the rest of the array. Each probe received a −log_10_ *p*-value score from the windowed Kolmogorov–Smirnov test around that probe. If several adjacent probes rose significantly above a *p*-value minimum cutoff (−log_10_) of 2, the region was considered to be an enrichment peak. When comparing two groups’ differentially enriched regions, we averaged the log_2_-ratio values for each group (Experiment and Control) and calculated M′ value for each probe, *M*′ = *Average*(log_2_ *MeDIP*_*E*_/*Input*_*E*_) − *Average*(log_2_ *MeDIP*_*C*_/*Input*_*C*_). Then the NimbleScan sliding-window peak-finding algorithm was rerun on these data to find the differential enrichment peaks.

In this study, hypermethylation region was defined as a region with log_2_-ratio value ≥0.585 and peak score ≥2, and hypomethylation region was defined as a region with log_2_-ratio value ≤–1 and peak score ≥2. The differentially methylated regions (DMRs) that were located in promoter regions and completely overlapped with CpG islands (CGI) were identified as candidate DMRs. Gene ontology (GO) analysis and Pathway analysis were performed to determine the roles of the candidate DMRs’ corresponding genes in biological pathways or identify GO terms using MAS 3.0 (http://bioinfo.capitalbio.com/mas3/).

### Gene expression microarray

Gene expression profiling was performed on the same 10 paired samples used in MeDIP-chip analysis by using the Agilent Oligo Microarray Kit 8×60 K (Agilent Technologies, Santa Clara, CA, USA) as described previously [5]. Significantly differentially expressed genes (DEGs) were identified by using the mixed model analysis of variance with a false discovery rate (Benjamini–Hochberg test)–adjusted *q*-value of ≤0.05 and absolute fold-change values ≥2. Hierarchical clustering was carried out using cluster 3.0. GO analysis and Pathway analysis were performed to determine the roles of DEGs.

### Integrated analysis

Integrated analysis of MeDIP-chip data and gene expression data consisted of the following steps. Step 1 was to identify candidate genes whose transcriptional repression is inversely related to promoter methylation. The genes whose DMRs were hypermethylated and whose expression was downregulated were identified as potential TSGs, and the genes whose DMRs were hypomethylated and whose expression was upregulated were identified as potential oncogenes. GO analysis and Pathway analysis were carried out to classify the functions of the candidate genes. Step 2 was to use localization and density of methylated CpG (mCpG) and promoter CpG content to select candidate genes. Genes which DMRs were located on the promoter region with high CpG content and high log2-ratio value were selected. Step 3 was to pinpoint candidate driver genes by retrieving gene function and research progress on NCBI (http://www.ncbi.nlm.nih.gov/pubmed); genes that had a documented potential role in tumorigenesis and had not been reported in lung cancer were screened out.

### Methylation-sensitive high-resolution melting assay

Methylation-sensitive high-resolution melting assay (MS-HRM) was performed on the 45 paired lung cancer and normal lung tissues to validate the methylation status of the candidate genes identified by the integrated analysis. A range of standards was included to control for bias in the sensitivity of the detection: 0% (unmethylated: EpiTect Control DNA; Qiagen), 100% (methylated: EpiTect Control DNA), and 50% (equal mixture of both templates). MS-HRM primers were designed to amplify a short amplicon size of less than 200 bp (http://www.urogene.org/cgi-bin/methprimer) and synthesized by Sangon Biotech (Shanghai, China) (S2 Table). MS-HRM assay was performed according to the instructions for the Epitect HRM PCR kit (Qiagen) on the RotorGene Q (Qiagen) in triplicate. A sample amplification curve between the 50% and 100% standard curves was defined as hypermethylation, and a sample amplification curve between the 0% and 50% standard curves was defined as hypomethylation.

### Quantitative DNA methylation analysis

Quantitative methylation analysis of DMRs in the candidate genes was performed on the 45 paired tumor and normal lung tissues by using the Sequenom MassARRAY platform (CapitalBio) as described previously [20]. This platform employs RNA base-specific cleavage and matrix-assisted laser desorption/ionization time-of-flight mass spectrometry. PCR primers for MassARRAY were designed by using Epidesigner (http://www.epidesigner.com) (S3 Table). The spectra methylation ratios were obtained via Epityper software version 1.0 (Sequenom, San Diego, CA, USA).

### Real-time fluorescent quantitative PCR

Real-time fluorescent quantitative PCR (RT-qPCR) was performed on 39 of the paired tumor and normal lung tissues to validate the mRNA expression of the candidate genes; the samples in the other 6 pairs were insufficient for this analysis. The analysis was carried out on an Applied Biosystems 7300 Real-Time PCR System (Applied Biosystems, Inc., Foster City, CA, USA) by using Quantinova SYBR Green PCR Master Mix (Qiagen). The relative expression values of tumor and normal lung tissues were calculated by the comparative Ct method. Primers for RT-qPCR are summarized in S4 Table. The expression of *β-actin* was used as a reference to normalize the other genes’ expression. Each experiment was performed in duplicate.

### Western blotting

Equal amounts of protein from each sample were separated by 12% sodium dodecyl sulfate–polyacrylamide gel electrophoresis (SDS-PAGE), transferred to polyvinylidene fluoride membranes (Merck Millipore, Billerica, MA, USA) by the semi-dry transfer protocol (Bio-Rad, Hercules, CA, USA), and blocked with 5% skimmed milk. The membranes were then probed with monoclonal antibodies against candidate genes and GAPDH overnight at 4°C. Antibody information is listed in S5 Table. The membranes were then rinsed with TBST and incubated with IgG conjugated to horseradish peroxidase at room temperature for 2 h. The protein bands were visualized on an ImageQuant LAS 500 (GE Healthcare, Pittsburgh, PA, USA) after applying electrochemiluminescent detection reagent (Millipore, Bedford, MA, USA). The intensities of the bands were quantified by using image J software. The band intensity for each candidate gene was subtracted from its own background intensity, normalized against the corresponding GAPDH intensity, and then compared with that of the paired sample. Each experiment was performed in duplicate.

### Statistical analysis

The statistical analyses were performed with the IBM SPSS statistics 22.0 vision software (IBM, Armonk, NY, USA). The Student *t*-test or Related-samples Wilcoxon signal rank test was used to compare continuous variables and the χ^2^ check was used to compare differences in enumeration data. The Pearson correlation test was used for correlation analyses. The validation results for the genes identified by integrated analysis were compared with clinicopathologic characteristics of the lung cancer in Xuanwei patients (patient age and sex, smoking history, tumor stage, lymph node metastasis, tumor size) by the Independent samples Mann Whitney U test or Independent samples Kruskal Wallis test. Overall survival was calculated by the log-rank test, and the Kaplan-Meier method was used to generate survival curves. A *p-*value <0.05 was considered statistically significant.

## Results

### Identification of DMRs from paired patient samples

**Fig 1:**
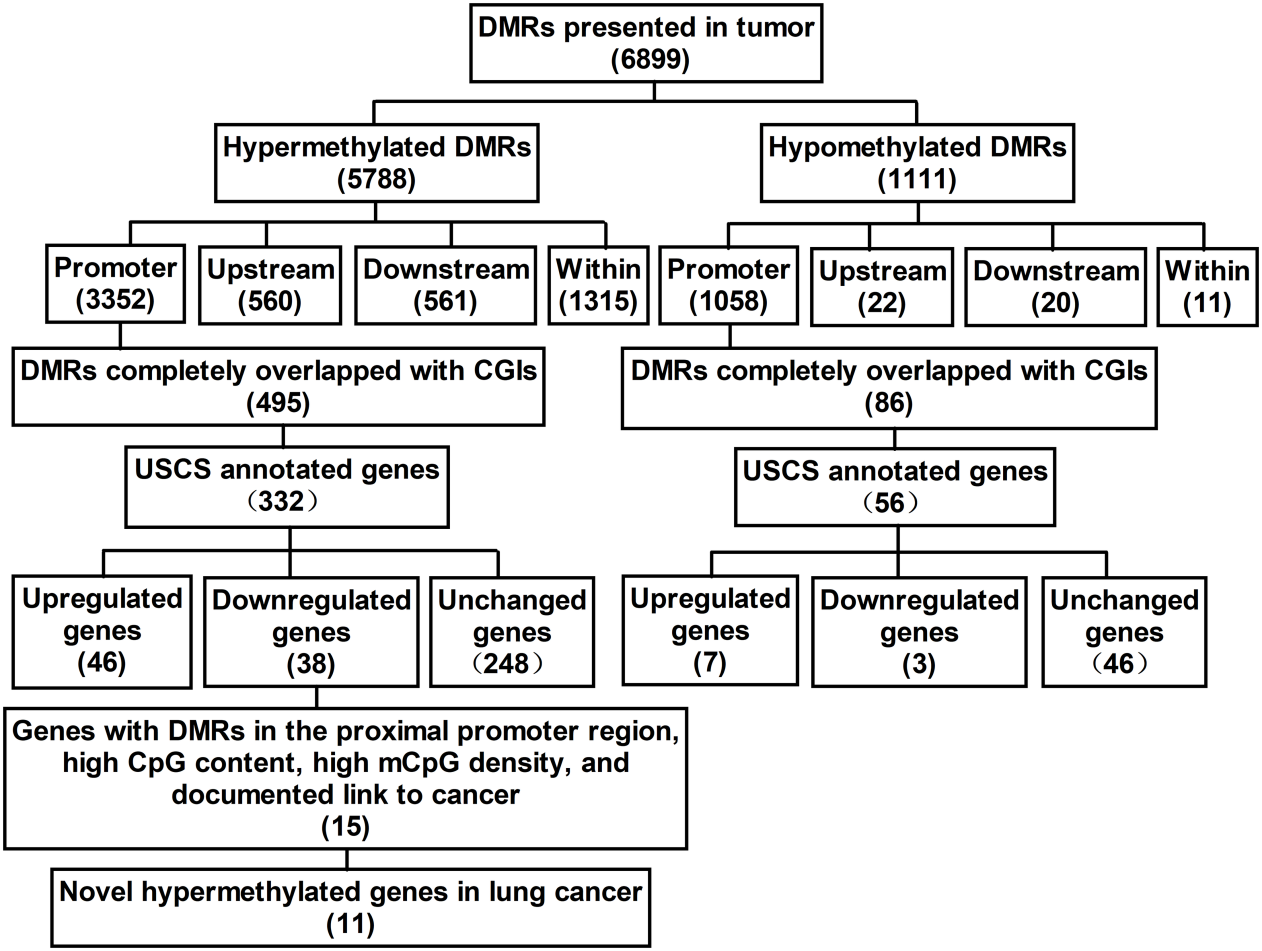
The flow chart of screening for methylated markers in lung cancer in Xuanwei. DMR, differentially methylated region; CGI, CpG island; mCpG, methylated CpG.

**Fig 2:**
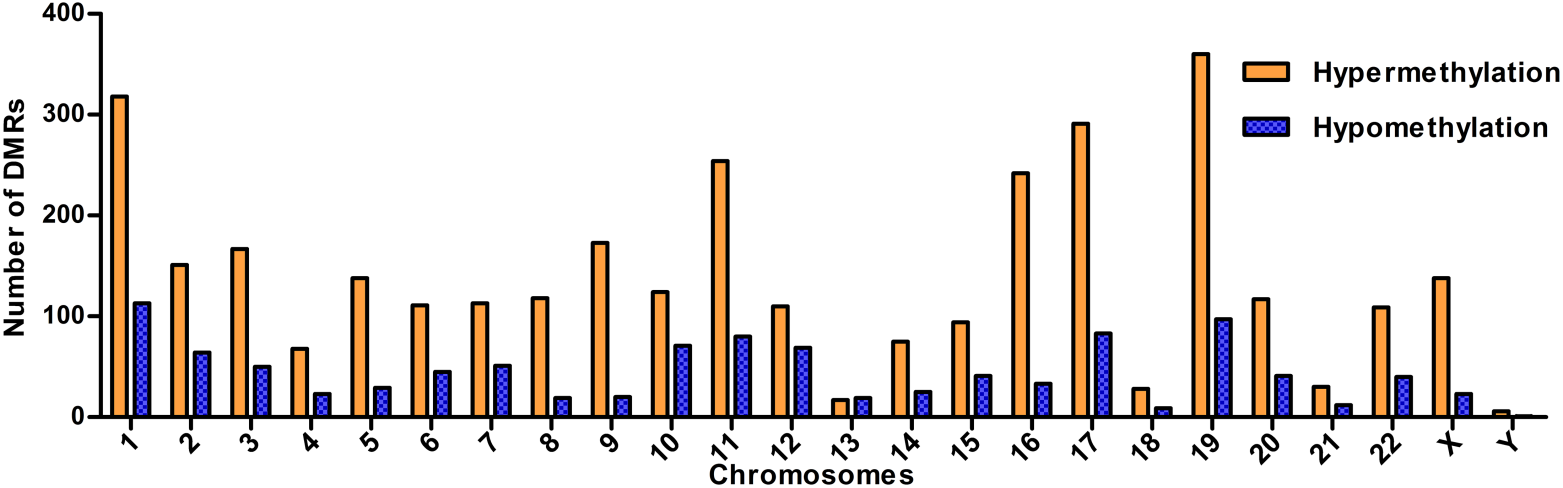
The distribution of 4,410 DMRs within promoter regions among the chromosomes.

MeDIP-chip analysis of genome-wide DNA methylation on paired lung cancer and normal lung tissues found that, compared with the normal tissues, the tumor tissues had a total of 6,899 DMRs, including 5,788 hypermethylated regions and 1,111 hypomethylated regions (the microarray data has uploaded to Gene Expression Omnibus (GEO), the relevant accession number is GSE113432). Of the hypermethylated regions, 560 were located in upstream regions of genes, 561 in downstream regions, 1,315 within genes, and 3,352 in promoter regions. Of the hypomethylated regions, 22 were located in upstream regions of genes, 20 in downstream regions, 11 within genes, and 1,058 in promoter regions (a flow chart of the steps in the analysis is presented in Fig 1). Thus, the majority of DMRs (63.8%) were located in promoter regions, mainly in chromosomes 19, 1, 17, and 11 (you can see it in Fig 2). The 581 DMRs (495 hypermethylated regions and 86 hypomethylated regions) located in promoter regions that completely overlapped with CGIs were screened out as candidate DMRs. A search for these candidate DMRs in the USCS Genome Browser identified 388 annotated potential tumor-specific methylation-altered genes (Fig 1). GO analysis indicated that these genes were involved in a wide range of important physiological processes, such as cellular processes, regulation of biological processes, catalytic activity, binding, biological regulation, metabolism, development, and transportation. Pathway analysis showed these genes were involved in many pathways, such as metabolic pathways, pathways in cancer, cAMP signaling pathway, and pathways involved in neuroactive ligand-receptor interaction, melanogenesis, HTLV-I infection, glutamatergic synapse, alcoholism, longevity, GABAergic synapse, PI3K-Akt signaling, calcium signaling, and estrogen signaling.

### Identification of DEGs from paired patient samples

Gene expression microarray analysis revealed significant differences in gene expression between the paired tumor and normal lung tissues. A total of 5,129 genes were identified as DEGs, whereas the other 11,861 genes were not differentially expressed. Of these DEGs, 3,248 were upregulated and the other 1,881 were downregulated. Cluster analysis of these genes showed a distinct separation between the gene expression of tumor and normal lung tissue (data not shown). GO analysis indicated that these DEGs were involved in a wide range of cancer-related processes, including cell division, cell adhesion, cell proliferation, and DNA replication. Pathway analysis showed these DEGs were involved in many pathways, including pathways involved in p53 signaling, MAPK, Jak-STAT signaling, hedgehog signaling, and non-small cell lung cancer.

### Integrated analysis of DMRs and DEGs

Integrated analysis of DMRs and DEGs identified 45 potential tumor-specific genes, 38 genes whose DMRs (58 DMRs) were hypermethylated and whose expression was downregulated in lung cancer and 7 genes whose DMRs (11 DMRs) were hypomethylated and whose expression was upregulated in tumor (Fig 1). It is interesting to note that the methylation level of some regions was also positively correlated to unique gene expression. GO analysis indicated that these genes were involved in a wide range of important physiological processes such as biological regulation, metabolism, development, and transportation. Pathway analysis showed these genes were involved in many pathways, including pathways involved in cancer, inflammatory mediator regulation of TRP channels, melanogenesis, neuroactive ligand-receptor interaction, chemokine signaling, and DNA replication. The genes with a DMR located on the proximal promoter, high CpG content in the promoter, high density of mCpG, and a documented link to cancer were then selected. Of these 15 genes (*STXBP6*, *MGAT3*, *BCL6B*, *FZD10*, *HSPB6*, *PF4V1*, *KLF4*, *PTGFR4*, *PDLIM2*, *LAMA4*, *ASPRV1*, *GATA2*, *HOXA2*, *LHX6*, and *KLF2*), 11 had not previously been reported in lung cancer (except *GATA2*, *HOXA2*, *LHX6*, and *KLF2*; Fig 1). Of these 11, 4 hypermethylated genes (*STXBP6*, *BCL6B*, *FZD10*, and *HSPB6*) were selected for further study.

### Validation of candidate gene methylation

**Fig 3:**
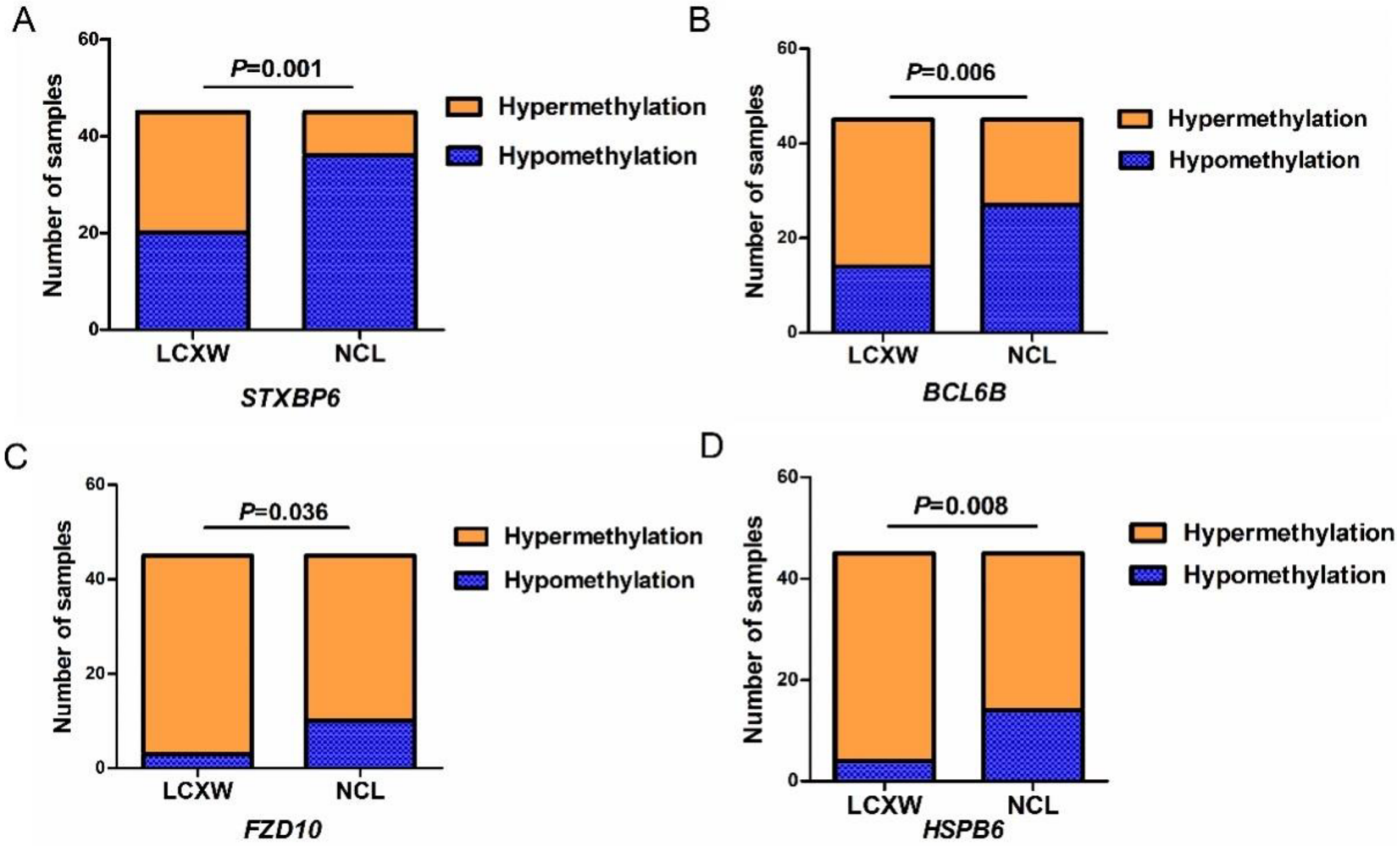
Methylation results in promoter of each candidate genes in 45 paired samples validated by MS-HRM. A, *STXBP6*; B, *BCL6B*; C, *FZD10*; D, *HSPB6*.

The promoter methylation of the 4 candidate genes was validated by MS-HRM and MassARRAY analyses on the 45 paired samples of lung cancer and noncancerous lung tissues, respectively. Supporting the MeDIP-chip data, statistically significant tumor-specific methylation was observed in all 4 candidate genes. The results of MS-HRM indicated that the hypermethylation ratios of the tested regions in the 4 candidate genes were significantly higher in the tumor than in the paired normal samples (*STXBP6*: 55.6% vs. 20%, *p* = 0.001; *BCL6B*: 68.9% vs. 40%, *p* = 0.006; *FZD10*: 93.3% vs. 77.8%, *p* = 0.036; *HSPB6*: 91.1% vs. 68.9%, *p* = 0.008) (the data is showed in Fig 3 and S6 Table).

**Fig 4:**
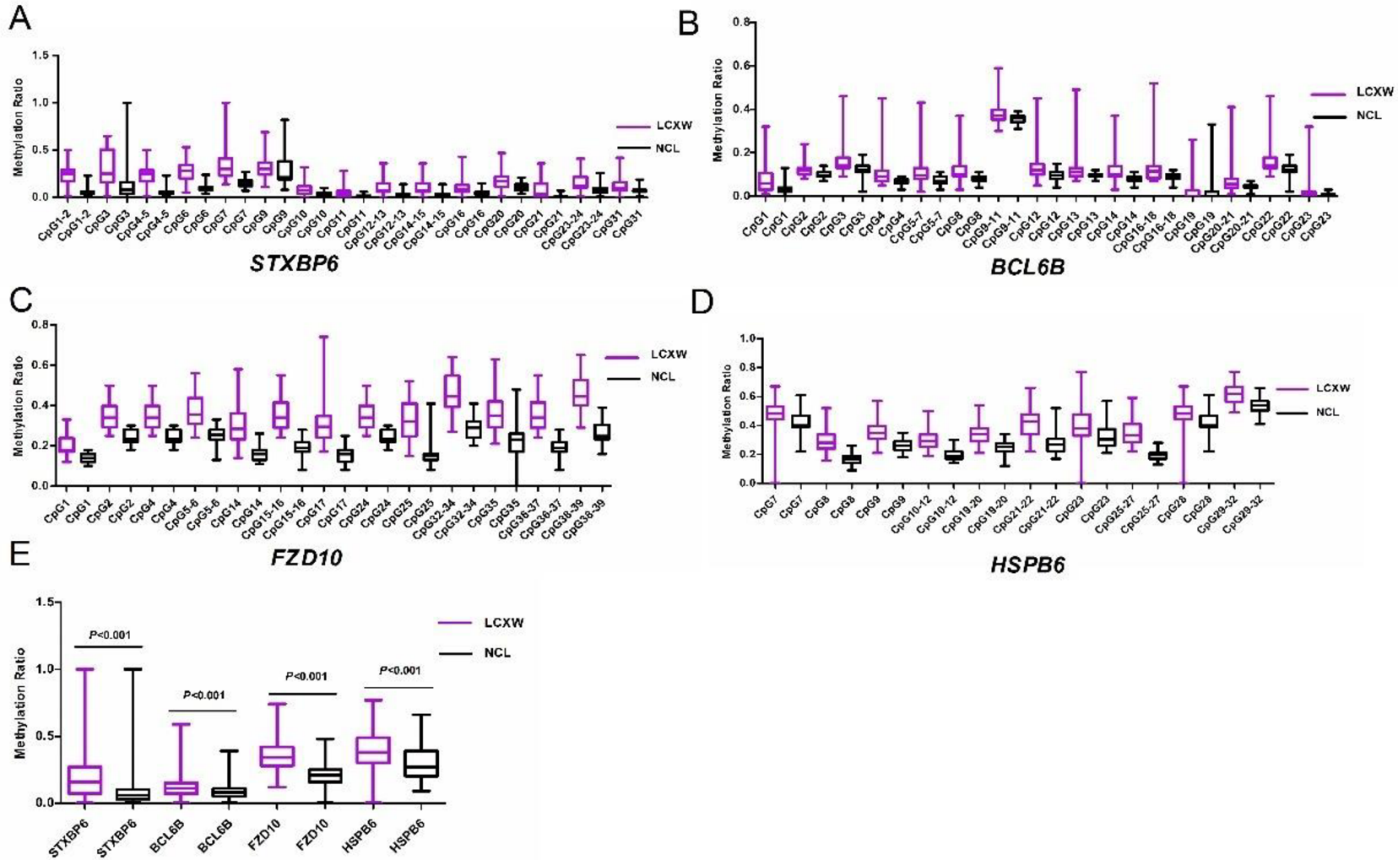
Methylation status of CpG units in the promoters of the 4 candidate genes detected by MassARRAY. (A-D) The individual genes: A, *STXBP6*; B, *BCL6B*; C, *FZD10*; D, *HSPB6*. (E) The overall methylation levels of the tested regions in each candidate genes.

Mass spectrometry analysis (MassARRAY) was performed to clarify the methylation status of CpG units in the promoters of the 4 candidate genes. The methylation ratio of a unit containing several CpG sites represents the average methylation of the CpG sites. In total, 15 of 19 units (20 of 31 CpG sites) in *STXBP6* promoter regions, 15 of 17 units (22 of 24 CpG sites) in *BCL6B* promoter regions, 13 of 18 units (20 of 39 CpG sites) in *FZD10* promoter regions, and 10 of 13 units (19 of 32 CpG sites) in *HSPB6* promoter regions were examined. CpG units undetected within the detected regions by this analysis were 8, 17–19, 22, and 25–30 in *STXBP6*; 15 and 24 in *BCL6B*; 3, 7, 8–13, 18–23, and 26–31 in *FZD10*; and 1–6, 13–18, and 24 in *HSPB6*. The results showed that the overall methylation levels of promoter regions in each genes were significantly higher in the tumor than in the paired normal tissues (Fig 4A–4E, the medians of the whole tested region of target genes in samples are showed in S7 Table), and the hypermethylation ratios of all CpG units except CpG 9 of *STXBP6* and CpG 9–11 of *BCL6B* were significantly higher in tumor than in paired normal tissues (S8 Table). The significantly methylated CpG units between tumor and normal lung tissues included CpG 1–2, 4–5, and 7 of *STXBP6*, CpG 4, 8, 14, and 16–18 of *BCL6B*, CpG 15–16, 25, 32–34, 36–37, and 38–39 of *FZD10*, and CpG 8, 21–22, and 25–27 of *HSPB6* (the median of each unit which above-mentioned of target genes in samples are showed in S7 Table).

### Validation of candidate gene expression

**Fig 5:**
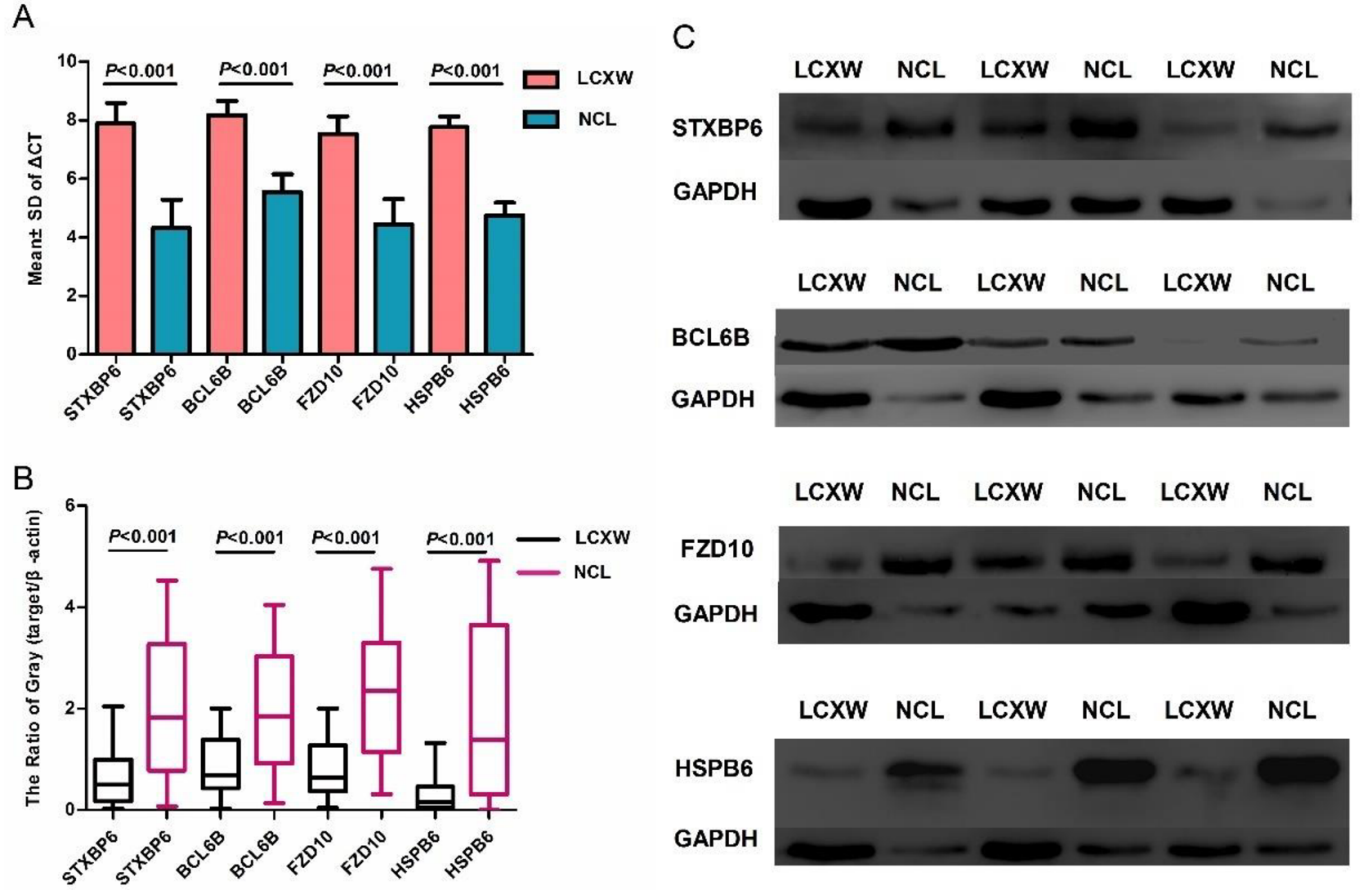
Expression of the 4 candidate genes in 39 paired samples. (A) Comparison of the mRNA expression levels of the 4 candidate genes between tumors and paired normal lung tissues. (B) Comparison of the expression level of the proteins encoded by the 4 candidate genes in tumors and paired normal lung tissues. (C) The gray level of the proteins encoded by the 4 candidate genes between tumors and paired normal lung tissues.

Hypermethylation of a promoter is likely to influence the expression of the gene. The expression of the 4 candidate genes was further validated by RT-qPCR and western blot on the 39 paired samples. Compared with paired normal tissues, both the mRNA and protein expression levels of the 4 candidate genes were significantly downregulated in most of the tumor tissues. *STXBP6* mRNA expression was downregulated in 66.67% of tumor tissues, *BCL6B* was downregulated in 69.23%, *FZD10* was downregulated in 71.79%, and *HSPB6* was downregulated in 74.36% (Fig 5A and S9 Table, *p* <0.001). STXBP6 protein expression was downregulated in 91.43% of tumor tissues, *BCL6B* was downregulated in 88.57%, *FZD10* was downregulated in 88.57%, and *HSPB6* was downregulated in 91.43% (Fig 5B and 5C and S10 Table, *p* <0.001, the original blots of western blots is shown in the S1–S8 Figs). Both RT-qPCR and western blot validated the inverse correlation between gene expression and overall methylation level of promoter regions; however, no inverse correlation was observed between gene expression and methylation of any single CpG unit in the promoters.

### Association of gene methylation and gene expression with clinicopathologic characteristics of lung cancer patients

**Fig 6:**
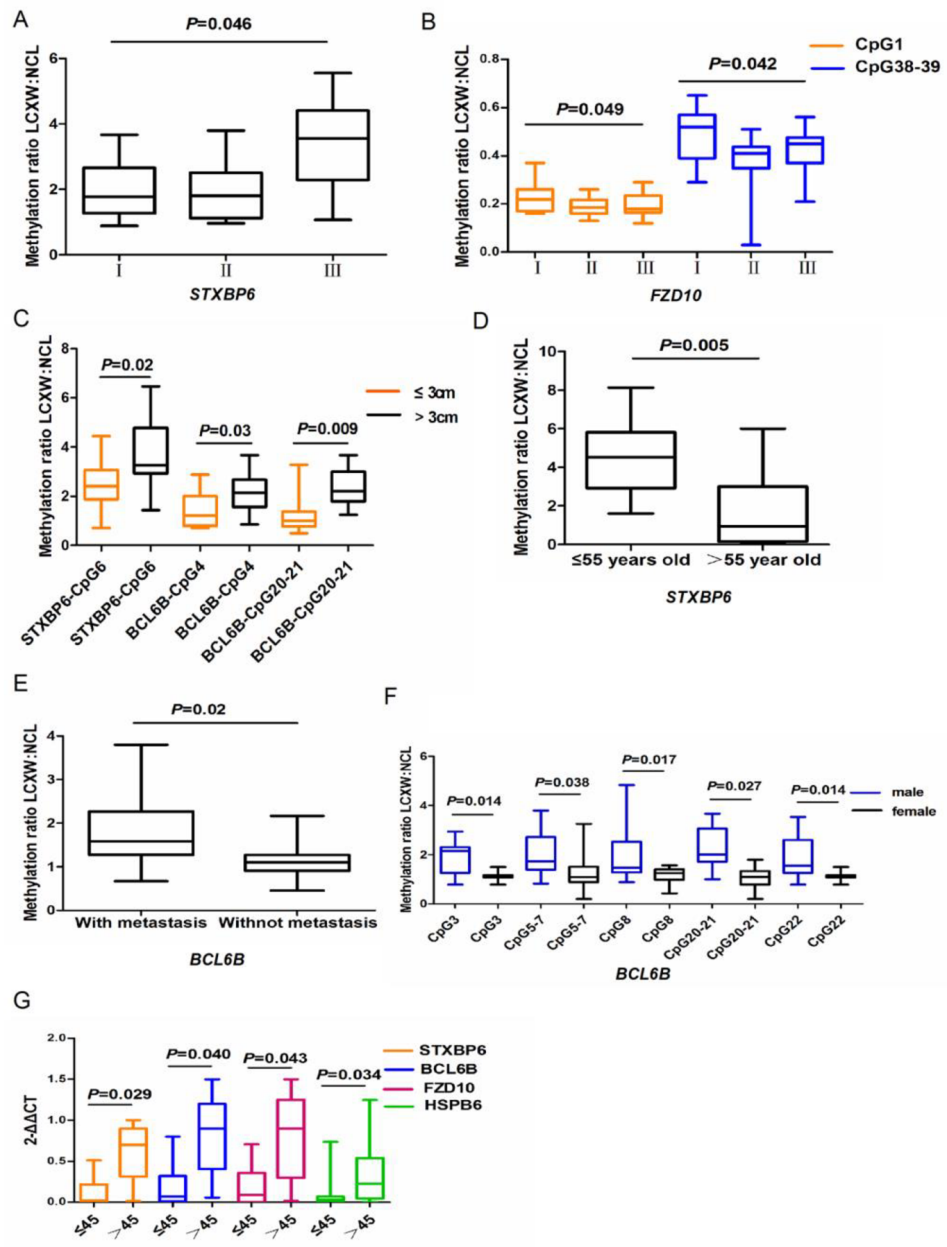
Correlation analysis the results of 4 candidate genes with the clinicopathologic characteristics of patients. (A) Methylation ratio of CpG site 7 of *STXBP6* grouped by lung cancer stage. (B) Methylation ratios of CpG sites 1 and 38 of *FZD10* grouped by lung cancer stage. (C) Methylation ratios of CpG site 6 of *STXBP6* and CpG sites 4 and 20 of *BCL6B* grouped by tumor size. (D) Methylation ratio of CpG site 3 of *STXBP6* grouped by patient age. (E) Methylation ratio of CpG site 12 of *BCL6B* grouped by lymph node metastasis status of patient. (F) Methylation ratios of CpG sites 3, 5, 8, 14, 20, and 22 of *BCL6B* grouped by patient sex. (G) Expression level of *mRNA* of the 4 candidate genes grouped by patient age.

Analysis of the association between gene methylation changes and clinicopathologic features showed that the methylation ratio of CpG unit 7 in *STXBP6* was significantly higher in stage III lung cancer than in early stage I and II lung cancer (Fig 6A, *p* <0.05); interestingly, the methylation ratios of CpG units 1 and 38–39 in *FZD10* were significantly higher in stage I lung cancer than in stage II and III lung cancer (Fig 6B, *p* <0.05); the methylation ratios of CpG unit 6 in *STXBP6* and CpG units 4 and 20–21 in *BCL6B* were significantly higher in patients whose cancer was >3 cm in diameter than in those whose tumor was <3 cm (Fig 6C, *p* <0.05); methylation of CpG unit 3 in *STXBP6* was significantly related to patient age: the methylation ratio was higher in patients younger than 55 years (Fig 6D, *p* = 0.005); the methylation ratio of CpG unit 12 in *BCL6B* was significantly higher in patients with lymph node metastasis than in those with no lymph node metastasis (Fig 6E, *p* = 0.02); and the methylation ratios of CpG units 3, 5–7, 8, 20–21, and 22 in *BCL6B* were significantly higher in male patients than in female patients (Fig 6F, *p* <0.05). It is important to note that the methylation ratio of CpG sites of these genes were no significant differences between smokers and non-smokers in this study (Data not shown). The expression of *STXBP6*, *BCL6B*, *FZD10*, and *HSPB6* mRNAs was significantly downregulated in patients younger than 45 years (Fig 6G, *p* = 0.029, *p* = 0.040, *p* = 0.043, *p* = 0.034, respectively), while no correlation was observed between the expression of the 4 candidate genes and other clinicopathologic features such as patient sex, smoking history, disease stage, or lymph node metastasis. Survival analysis showed there was no association between gene methylation change or differential gene expression and clinicopathologic features (Data not shown).

## Discussion

Lung cancer in Xuanwei provides a unique opportunity to research the pathogenesis of non–tobacco-related lung cancer. Our integrated analysis of genome-wide DNA methylation and gene expression in lung cancer identified a large number of abnormal methylation changed genes and revealed several genes regulated by promoter methylation that have not been described in lung cancer before, these results lay an important foundation for clarifying the carcinogenesis of lung cancer in Xuanwei and represent promising novel methylation markers for the diagnosis and therapy of lung cancer.

In this study, we pooled gDNA from 10 lung cancer and from their paired noncancerous lung samples into two groups and compared their methylation. The individual DMR variation between patients would be difficult to determine and may be of no significance, because random changes may occur in each patient. By sample pooling, the random individual difference among subjects was eliminated. While the sample pooling led to loss of individual detailed information, this wasn’t a concern for this study.

MeDIP-chip analysis identified a total of 6,899 tumor-specific DMRs, including 5,788 hypermethylated regions and 1,111 hypomethylated regions, in the tumor compared with the normal lung tissue, indicating that tumor-specific DMRs are widespread in lung cancer and that hypermethylated regions are significantly more frequent than hypomethylated regions (5.2:1) in these tumors. Both the genomic location and chromosome distribution of the DMRs were heterogeneous. Due to paired samples and strict cut-off values, in theory, all these DMRs could have contributed to the development and progression of lung cancer in Xuanwei.

Integrated analysis of DMRs and DEGs identified 45 tumor-specific candidate genes. And then these candidate genes were compared with some results of similar study. Top 25 differentially methylated loci in promoters and negatively correlated with expression in 16 lung adenocarcinomas tissues using a methylation-sensitive restriction enzyme- based HELP microarray assay were found in Nandita Mullapudi’s study [19], *NQO1* was appeared with hypomethylation and upregulated expression in Nandita Mullapudi’s [21] and this study. In Maria Moksnes Bjaanæs’ study [22], it determined whole-genome DNA methylation profiles of 164 fresh frozen lung adenocarcinoma samples and 19 samples of matched normal lung tissue using the Illumina Infinium 450K array, it found methylation level of 671(68.6%) gene regions were negatively correlated to 520 unique genes expression. These genes compared with the 45 candidate genes, *PTGER4*, *ICAM4*, *AIM2* were negatively correlated between the level of methylation and gene expression in 2 study, but the direction is the opposite. These results suggest that the same methylation change in different research is very rare, but a lot of methylation changes have taken place in lung adenocarcinoma in almost all study, genome-wide methylation trends were same in roughly [21–22], differences of methylation level of the same gene in difference study may be caused by different races, the types of tumors and environment. So, it is necessary to research lung cancer in Xuanwei.

4 hypermethylated genes including *STXBP6*, *BCL6B*, *FZD10* and *HSPB6* that had not been reported in lung cancer were selected for further study. The expression of these 4 hypermethylated genes was significantly downregulated in most of tumor samples suggests that methylation is responsible for regulating expression of these genes and that they may be novel methylation markers in lung cancer in Xuanwei. Our observed upregulation of hypermethylated genes and downregulation of hypomethylated genes in a minority of these cases may be explained by other regulatory mechanisms for gene expression, such as noncoding RNA regulation and histone modification.

*STXBP6* (syntaxin binding protein 6) is located on 14q12 and encodes a factor binding components of the SNARE complex that has a central role in vesicle targeting and fusion in eukaryotic cells. To date, *STXBP6* has not been reported in human cancer except in breast cancer cell transformation [23]. In the in vitro model of breast cancer progression used in that study, *STXBP6* was hypermethylated and downregulated in the transformed cells compared to the parental MCF-10F cells. Treatment of transformed cells with the demethylating agent 5-aza-dC alone or in combination with the histone deacetylase inhibitor trichostatin increased *STXBP6* expression, which suggested that its downregulation, mediated by DNA methylation, may play an important role in MCF10F transformation [23]. In our study, *STXBP6* was significantly hypermethylated and downregulated in most of the tumor samples, and the hypermethylation ratios of CpG units 3, 6, and 7 in *STXBP6* were closely associated with patient age, tumor size, and tumor stage, respectively, suggesting that abnormal methylation in these CpG units of the *STXBP6* promoter might serve as novel methylation markers for diagnosis of lung cancer in Xuanwei.

*BCL6B* (B cell CLL/lymphoma 6 member B) is a homologue of B cell CLL/lymphoma 6 (*BCL6*) located on chromosome 17p13.1. *BCL6B* is regarded as a potential tumor suppressor, and methylation of *BCL6B* is a marker of poor prognosis in several human cancers, including gastric cancer [24–26], hepatocellular carcinoma [27, 28], and colorectal cancer [29]. *BCL6B* expression was significantly decreased in these cancer tissues compared with paired normal tissues because of promoter methylation. Re-expression of *BCL6B* in gastric cancer, hepatocellular carcinoma, and colorectal cancer cell lines can inhibit colony formation, suppress cell viability, induce apoptosis, and restrain their tumorigenicity in nude mice [27–29]. Stable expression of *BCL6B* suppressed migration and invasion of MHCC97L cells and significantly reduced the incidence and severity of lung metastasis in an orthotopic mouse model of hepatocellular carcinoma [30]. Epigenetic silencing of *BCL6B* can inactivate p53 signaling and cause hepatocellular carcinoma and colorectal cancer cells to become resistant to 5-fluorouracil [28, 29]. In our study, methylation of some CpG units in the *BCL6B* promoter region was associated with clinicopathologic characteristics of tumor patients, including tumor size, lymph node metastasis, and patient sex. These results suggest that *BCL6B* hypermethylation promotes tumor onset and progression; these abnormally methylated CpG units in the *BCL6B* promoter region might serve as novel methylation markers for diagnosis of the lung cancer.

*FZD10* (frizzled class receptor 10), located on chromosome 12q24.33, is a member of the frizzled gene family and encodes a cell-surface receptor for molecules in the Wnt pathway. *FZD10* is highly upregulated in several cancers and cancer cells, including synovial sarcoma [31], primary colon cancer [32], cervical cancer cells [30], and glioblastoma cells [32], and may play critical roles in the development/progression of human cancers by various molecular mechanisms. *FZD10* has been implicated in carcinogenesis by its positive regulation of the WNT-β-catenin-TCF signaling pathway [32] and its activation of the non-canonical Dvl-Rac1-JNK pathway [33]. BRMS1L-induced *FZD10* epigenetic silencing through HDAC1 recruitment and histone H3K9 deacetylation at the promoter can suppress breast cancer metastasis by inhibiting aberrant activation of WNT3-FZD10-β-catenin signaling [34]. Colorectal cancer cells immunopositive for FZD10, however, showed significantly less nuclear accumulation of β-catenin than FZD10-immunonegative cells, and there was a strong inverse correlation between nuclear immunostaining scores for β-catenin expression and expression patterns of *FZD10*, suggesting that *FZD10* has a distinct role in canonical Wnt signal transduction [35]. To our surprise, *FZD10* was significantly hypermethylated and downregulated in a majority of the tumor tissues, and the methylation ratios of CpG units 1 and 38–39 in the *FZD10* promoter were significantly higher in stage I lung cancer than in stage II and III disease. *FZD10* may play different roles in different types of cancers, and *FZD10* methylation may be an early event in the development of lung cancer; both ideas are worthy of further study.

*HSPB6* (heat shock protein family B [small] member 6), also referred to as *HSP20*, is located on chromosome 19q13.12 and encodes a small heat shock protein that is ubiquitously expressed in various tissues and has several functions. Elevated HSP20 expression can promote myocardial angiogenesis through activation of the VEGFR signaling cascade and protect the heart against various stress stimuli [36]. *HSP20* may play a protective role against the development and progression of various human cancers and may represent a new target for the prediction and treatment of these cancers [37–42]. *HSP20* was significantly hypermethylated and downregulated in melanomas compared to normal melanocytes [43]. Expression of *HSP20* was inversely correlated with tumor progression in patients with ovarian cancer [40]. Upregulation of *HSP20* in human hepatocellular carcinoma cells can inhibit cell proliferation via suppression of the AKT signaling pathway [42], inhibit TGFα-induced migration and invasion via suppression of the JNK signaling pathway [37], and stimulate the caspase cascade through direct association with Bax [41]; *HSP20* upregulation also suppresses hepatocellular carcinoma progression by downregulating TNFα-stimulated intracellular signaling [38]. Downregulation of *HSP20* was observed in colorectal cancer specimens compared with their paired normal tissues and was related to advanced TNM stage, lymph node metastasis, and tumor recurrence [39]. Overexpression of *HSP20* in HCT-116 human colorectal cancer cells induced massive apoptosis in a time-dependent manner by enhancing caspase-3/7 activity and downregulating mRNA and proteins levels of anti-apoptotic Bcl-xL and Bcl-2 [39]. In our study, *HSPB6* was significantly hypermethylated and downregulated in most of the tumor tissues, suggesting that it plays a protective role in the onset of lung cancer in Xuanwei.

In summary, this genome-wide integrative analysis of DNA methylation and gene expression in tumor identified novel DNA methylation markers that are related to lung cancer in Xuanwei. Despite the small number of samples, our data show that the incidence of aberrant promoter methylation increased significantly during tumor development. The aberrantly methylated promoters and CGIs reported here provide new insight into the carcinogenesis of lung cancer in Xuanwei and represent a promising avenue for further investigation as novel diagnostic and therapeutic targets.

## Supporting information

S1 Table Clinicopathologic characteristics of 45 patients. (PDF)

S2 Table Primers used for MS-HRM. (PDF)

S3 Table Primers used for MassARRAY. (PDF)

S4 Table Primers used for RT-qPCR. (PDF)

S5 Table Antibodies used for western blot analysis. (PDF)

S6 Table Comparison of promoter hypermethylation of the 4 candidate genes in lung cancer and normal lung tissues by MS-HRM. (PDF)

S7 Table The medians of methylation level of promoter regions in the 4 genes in lung cancer and normal lung tissues by MassARRAY. (PDF)

S8 Table Comparison of promoter hypermethylation of the 4 candidate genes in lung cancer and normal lung tissues by MassARRAY. (PDF)

S9 Table Comparison of mRNA expression of 4 candidate genes in lung cancer and normal lung tissues by RT-qPCR. (PDF)

S10 Table Comparison of expression of proteins encoded by 4 candidate genes in lung cancer and normal lung tissues by western blot. (PDF)

S1 Fig The original image of western blot of *STXBP6*. The image shows the blots of 9–24 paired samples, the first sample is cancer, the second one is the paired normal lung tissue, and so on. The first stripe is one in Fig 5C.
(TIFF)

S2 Fig The original image of western blot of *GAPDH*. The image shows the blots of 9–24 paired samples, the first sample is cancer, the second one is the paired normal lung tissue, and so on. The first stripe is one in Fig 5C and related to S1 Fig.
(TIFF)

S3 Fig The original image of western blot of *BCL6B*. (TIFF)

S4 Fig The original image of western blot of *GAPDH*. The image shows the blots of 9–24 paired samples, the first sample is cancer, the second one is the paired normal lung tissue, and so on. The first stripe is one in Fig 5C and related to S3 Fig.
(TIFF)

S5 Fig The original image of western blot of *FZD10*. The image shows the blots of 9–24 paired samples, the first sample is cancer, the second one is the paired normal lung tissue, and so on. The fourth stripe is one in Fig 5C.
(TIFF)

S6 Fig The original image of western blot of *GAPDH*. The image shows the blots of 9–24 paired samples, the first sample is cancer, the second one is the paired normal lung tissue, and so on. The fourth stripe is one in Fig 5C and related to S5 Fig.
(TIFF)

S7 Fig The original image of western blot of *HSBP6*. The image shows the blots of 9–24 paired samples, the first sample is cancer, the second one is the paired normal lung tissue, and so on. The second stripe is one in Fig 5C.
(TIFF)

S8 Fig The original image of western blot of *GAPDH*. The image shows the blots of 9–24 paired samples, the first sample is cancer, the second one is the paired normal lung tissue, and so on. The second stripe is one in Fig 5C and related to S7 Fig.
(TIFF)

## Abbreviations

DMRs: Differentially methylated regions
DEGs: Differentially expressed genes
MeDIP-chip: Methylated DNA immunoprecipitation combined with microarray
CpG: Cytosine–guanine
CGI: CpG islands
GO: Gene ontology
mCpG: methylated CpG
MS-HRM: Methylation-sensitive high-resolution melting
RT-qPCR: Real-time fluorescent quantitative PCR
SDS-PAGE: Sodium dodecyl sulfate–polyacrylamide gel electrophoresis
