# Supplementary material for: Integrated analysis of DNA methylation profiling and gene expression profiling identifies novel markers in lung cancer in Xuanwei, China
Source: PLoS One. 2018 Oct 4;13(10):e0203155. doi: 10.1371/journal.pone.0203155 (PMC6171826; doi:10.1371/journal.pone.0203155)
Supplement: S1 Table — (PDF) [file pone.0203155.s001.pdf]

Supplemental Table S1. Clinicopathologic characteristics of 45 patients.

Characteristic		Number of patients (%)
Sex	Male	28/45 (62.2%)
	Female	17/45 (37.8%)
Age (years)	≤45	15/45 (33.3%)
	≥46	30/45 (66.7%)
Smoking history	Yes	21/45 (46.7%)
	No	24/45 (53.3%)
Pathologic stage	I	23/45 (51.1%)
	II	12/45 (26.7%)
	III	10/45 (22.2%)
Pathologic types	adenocarcinoma	45/45(100%)
Lymph node metastasis	Yes	24/45 (53.3%)
	No	21/45 (46.7%)
Tumor size (cm)	0-3	21/45 (46.7%)
	3.1-5	21/45 (46.7%)
	≥5.1	3/45 (6.7%)
Survival duration (months)	1-12	9/45 (20%)
	13-24	16/45 (35.6%)
	25+	20/45(44.4%)
