# Supplementary material for: Integrated analysis of DNA methylation profiling and gene expression profiling identifies novel markers in lung cancer in Xuanwei, China
Source: PLoS One. 2018 Oct 4;13(10):e0203155. doi: 10.1371/journal.pone.0203155 (PMC6171826; doi:10.1371/journal.pone.0203155)
Supplement: S2 Table — (PDF) [file pone.0203155.s002.pdf]

Supplemental Table S2. Primers used for MS-HRM.

Gene	Primer sequence	Production size	Annealing temperature	Number of CpG
STXBP6	F:TTCGTGTTTTTGGTTTTTTTTAATTT	161	57°C	13
	R:ACCCCGAACTCCCCCACAC			
BCL6B	F:TTTACGGTTTTTATAGGTTTGTGTC	185	59°C	19
	R:TATATACTCTAAAAAATTACCCGCC			
FZD10	F:TGTCGGAAATTTTTTAATAAGAACG	200	58°C	23
	R:TAATAAAACTTACCCGAATTATCGC			
HSPB6	F: TTAATTTCGGAGGTTAGAGTGAGTC	156	57°C	15
	R:CTAACAACACCGAAAAATAACCG			

F, forward; R, reverse.
