# Supplementary material for: Integrated analysis of DNA methylation profiling and gene expression profiling identifies novel markers in lung cancer in Xuanwei, China
Source: PLoS One. 2018 Oct 4;13(10):e0203155. doi: 10.1371/journal.pone.0203155 (PMC6171826; doi:10.1371/journal.pone.0203155)
Supplement: S3 Table — (PDF) [file pone.0203155.s003.pdf]

Supplemental Table S3. Primers used for MassARRAY.

Gene	Primer sequence	Production sequence	Production sequence of MS-HRM	Total CpG unit	Detected CpG unit
STXBP6	F:ggaagagagTTGGTAGTAATGATTTTTTTAGAAGATGT<br>R:cagtaatacgactcactatagggagaaggctAAACCAAAAAAATAAACAAACTCCC	CHRO 14 -: 24588428-24588806	CHRO14-: 24588517-24588699	19	15
BCL6B	F:aggaagagagGGTTGGAAGTTTTGGTTTTAGTTGT<br>R:cagtaatacgactcactatagggagaaggctACCCTATAATCCCACCCCTTTC	CHRO 17+: 6867596-6867950	CHRO17+: 6867687-6867871	17	15
FZD10	F:aggaagagagGATTATGGAGTAGTTTAATTTTAAGTGGT<br>R:cagtaatacgactcactatagggagaaggctCCCAAATAACCAACCAAACC	CHRO12+: 129213797-129214143	CHRO12+: 129213840-129214039	18	13
HSPB6	F:aggaagagagGTGTTTATTTTGGAGGGGAGG<br>R:cagtaatacgactcactatagggagaaggctATAAATCCTAAAACCTAACCCAACC	CHRO19-: 40938416-40938694	CHRO19-: 40938593-40938754	13	10

F, forward; R, reverse.
