# Supplementary material for: Integrated analysis of DNA methylation profiling and gene expression profiling identifies novel markers in lung cancer in Xuanwei, China
Source: PLoS One. 2018 Oct 4;13(10):e0203155. doi: 10.1371/journal.pone.0203155 (PMC6171826; doi:10.1371/journal.pone.0203155)
Supplement: S4 Table — (PDF) [file pone.0203155.s004.pdf]

Supplemental Table S4. Primers used for RT-qPCR.

Gene	Primer sequence	Production size	Annealing temperature
STXBP6	F:GATGCCCCCTTTCCGATCTC	126	60°C
	R:ACGCAGTGAATCTACTCCAGC		
BCL6B	F:GCTTCATCCAGGCCAGCTAT	154	60°C
	R:CCTTGACTGCAGCTTCGAGA		
FZD10	F:GCTCAAGTGCTCCCCGATTA	108	60°C
	R:GCCTCCATGCACAGGTAGTT		
HSPB6	F:TTTCGGTGCTGCTAGACGTG	116	60°C
	R:GAATCCGTGCTCATCCGGG		
β-actin	F:CTGGGACGACATGGAGAAAA	564	60°C
	R:AAGGAAGGCTGGAAGAGTGC		

F, forward; R, reverse.
