# Supplementary material for: Integrated analysis of DNA methylation profiling and gene expression profiling identifies novel markers in lung cancer in Xuanwei, China
Source: PLoS One. 2018 Oct 4;13(10):e0203155. doi: 10.1371/journal.pone.0203155 (PMC6171826; doi:10.1371/journal.pone.0203155)
Supplement: S5 Table — (PDF) [file pone.0203155.s005.pdf]

Supplemental Table S5. Antibodies used for western blot analysis.

**Antibody**	**Manufacturer**	**Dilution**
Monoclonal rabbit anti-human STXBP6	Abcam, Cambridgeshire, UK	1:400
Monoclonal rabbit anti-human BCL6B	GeneTex, TA, USA	1:2000
Monoclonal rabbit anti-human FZD10	GeneTex, TA, USA	1:2000
Monoclonal Mouse anti-human HSPB6	R & D, MN, USA	1:1000
Monoclonal chicken anti-human GAPDH	Merck Millipore, MA,USA	1:2000
Peroxidase-conjugated goat anti-rabbit IgG(H+L)	Merck Millipore, MA,USA	1:5000
Peroxidase-conjugated goat anti-mouse IgG(H+L)	Merck Millipore, MA,USA	1:5000
Peroxidase-conjugated goat anti-chicken IgG (H+L)	KPL, MD, USA	1:5000
