# Supplementary material for: Integrated analysis of DNA methylation profiling and gene expression profiling identifies novel markers in lung cancer in Xuanwei, China
Source: PLoS One. 2018 Oct 4;13(10):e0203155. doi: 10.1371/journal.pone.0203155 (PMC6171826; doi:10.1371/journal.pone.0203155)
Supplement: S6 Table — (PDF) [file pone.0203155.s006.pdf]

Supplemental Table S6. Comparison of promoter hypermethylation of the 4 candidate genes in lung cancer and normal lung tissues by MS-HRM.

**Gene**	**Ratio of hypermethylation in lung cancer**	**Ratio of hypermethylation in noncancerous tissue**	**Pearson χ2**	***p* (2 sided)**
STXBP6	25/45(55.60%)	9/45(20.00%)	12.10	0.001
BCL6B	31/45(68.90%)	18/45(40.00%)	7.57	0.006
FZD10	42/45(93.30%)	35/45(77.80%)	4.41	0.036
HSPB6	41/45(91.10%)	31/45(68.90%)	6.94	0.008
