# Supplementary material for: Integrated analysis of DNA methylation profiling and gene expression profiling identifies novel markers in lung cancer in Xuanwei, China
Source: PLoS One. 2018 Oct 4;13(10):e0203155. doi: 10.1371/journal.pone.0203155 (PMC6171826; doi:10.1371/journal.pone.0203155)
Supplement: S7 Table — (PDF) [file pone.0203155.s007.pdf]

Supplemental Table S7. The medians of methylation level of promoter regions in the 4 genes in lung cancer and normal lung tissues by MassARRAY.

**Gene**	**CpG unit**	**Median of tumor**	**Median of normal**	***p* (2 tailed)**
STXBP6	1-2	0.25	0.05	<0.001
	4-5	0.25	0.04	<0.001
	7	0.32	0.14	<0.001
	The whole tested unit	0.16	0.06	<0.001
BCL6B	4	0.09	0.07	<0.001
	8	0.10	0.08	<0.001
	14	0.10	0.08	<0.001
	16-18	0.12	0.09	<0.001
	The whole tested unit	0.11	0.08	<0.001
FZD10	15-16	0.34	0.19	<0.001
	25	0.32	0.15	<0.001
	32-34	0.45	0.29	<0.001
	36-37	0.34	0.19	<0.001
	38-39	0.45	0.25	<0.001
	The whole tested unit	0.34	0.21	<0.001
HSPB6	8	0.28	0.17	<0.001
	21-22	0.43	0.27	<0.001
	25-27	0.34	0.20	<0.001
	The whole tested unit	0.38	0.27	<0.001
