# Supplementary material for: Integrated analysis of DNA methylation profiling and gene expression profiling identifies novel markers in lung cancer in Xuanwei, China
Source: PLoS One. 2018 Oct 4;13(10):e0203155. doi: 10.1371/journal.pone.0203155 (PMC6171826; doi:10.1371/journal.pone.0203155)
Supplement: S8 Table — (PDF) [file pone.0203155.s008.pdf]

**Supplemental Table S8**. Comparison of promoter hypermethylation of the 4 candidate genes in lung cancer and normal lung tissues by MassARRAY.

| **Gene** | **CpG unit** | **Statistical method** | ***p* (2 tailed)** |
|---|---|---|---|
| *STXBP6* | 1-2,4-5,6,10 | Paired samples test | <0.001 |
| | 3,7, 11,12-13,14-15,16,20,21,23-24,31 | Related-samples Wilcoxon signal rank test | <0.001 |
| | 9 | Related-samples Wilcoxon signal rank test | =0.058 |
| | The whole tested unit | Related-samples Wilcoxon signal rank test | <0.001 |
| *BCL6B* | 1,2,3,4,5-7,8,12,13,14,16-18,19,20-21,22,23 | Related-samples Wilcoxon signal rank test | <0.01 |
| | 9-11 | Related-samples Wilcoxon signal rank test | =0.759 |
| | The whole tested unit | Related-samples Wilcoxon signal rank test | <0.001 |
| *FZD10* | 1,2,4,5-6,14,15-16,24,25,32-34,35,36-37,38-39 | Paired samples test | <0.001 |
| | 17 | Related-samples Wilcoxon signal rank test | <0.001 |
| | The whole tested unit | Paired samples test | <0.001 |
| *HSPB6* | 8,9,19-20,21-22,25-27,29-32 | Paired samples test | <0.001 |
| | 7,10-12,23,28 | Related-samples Wilcoxon signal rank test | <0.001 |
| | The whole tested unit | Related-samples Wilcoxon signal rank test | <0.001 |
