# Supplementary material for: Integrated analysis of DNA methylation profiling and gene expression profiling identifies novel markers in lung cancer in Xuanwei, China
Source: PLoS One. 2018 Oct 4;13(10):e0203155. doi: 10.1371/journal.pone.0203155 (PMC6171826; doi:10.1371/journal.pone.0203155)
Supplement: S9 Table — (PDF) [file pone.0203155.s009.pdf]

Supplemental Table S9. Comparison of mRNA expression of 4 candidate genes in lung cancer and normal lung tissues by RT-qPCR.

**Gene**	**Mean ± SD** **(ΔCT_{tumor})**	**Mean ± SD** **(ΔCT_{normal})**	**Statistical method**	**T value**	***p*(2 tailed)**	**Down/total**
STXBP6	7.89±4.32	4.29±3.04	Paired samples test	T=5.13	＜0.001	66.67% (26/39)
BCL6B	8.18±3.04	5.56±3.79	Paired samples test	T=5.29	＜0.001	69.23% (27/39)
FZD10	7.54±3.74	4.44±2.44	Paired samples test	T=5.37	＜0.001	71.79% (28/39)
HSPB6	7.77±2.26	4.75±2.72	Paired samples test	T=6.83	＜0.001	74.36% (29/39)

Down/total: number of downregulated samples/total samples
