# Supplementary material for: Integrated analysis of DNA methylation profiling and gene expression profiling identifies novel markers in lung cancer in Xuanwei, China
Source: PLoS One. 2018 Oct 4;13(10):e0203155. doi: 10.1371/journal.pone.0203155 (PMC6171826; doi:10.1371/journal.pone.0203155)
Supplement: S10 Table — (PDF) [file pone.0203155.s010.pdf]

Supplemental Table S10. Comparison of expression of proteins encoded by 4 candidate genes in lung cancer and normal lung tissues by western blot.

**Protein**	**Median gray ratio in tumor**	**Median gray ratio in normal**	**Statistical method**	***P* (2 tailed)**	**Down/total**
STXBP6	0.61	1.83	Related-samples Wilcoxon signal rank test	＜0.001	91.43% (32/35)
BCL6B	0.79	1.89	Related-samples Wilcoxon signal rank test	＜0.001	88.57% (31.35)
FZD10	0.64	2.36	Related-samples Wilcoxon signal rank test	＜0.001	88.57% (31.35)
HSPB6	0.16	1.38	Related-samples Wilcoxon signal rank test	＜0.001	91.43% (32/35)

Gray ratio: the gray of target protein/the gray of GAPDH

Down/total: number of downregulated samples/total samples
