# Supplementary figures and images for: Integrated analysis of DNA methylation profiling and gene expression profiling identifies novel markers in lung cancer in Xuanwei, China
Source: PLoS One. 2018 Oct 4;13(10):e0203155. doi: 10.1371/journal.pone.0203155 (PMC6171826; doi:10.1371/journal.pone.0203155)

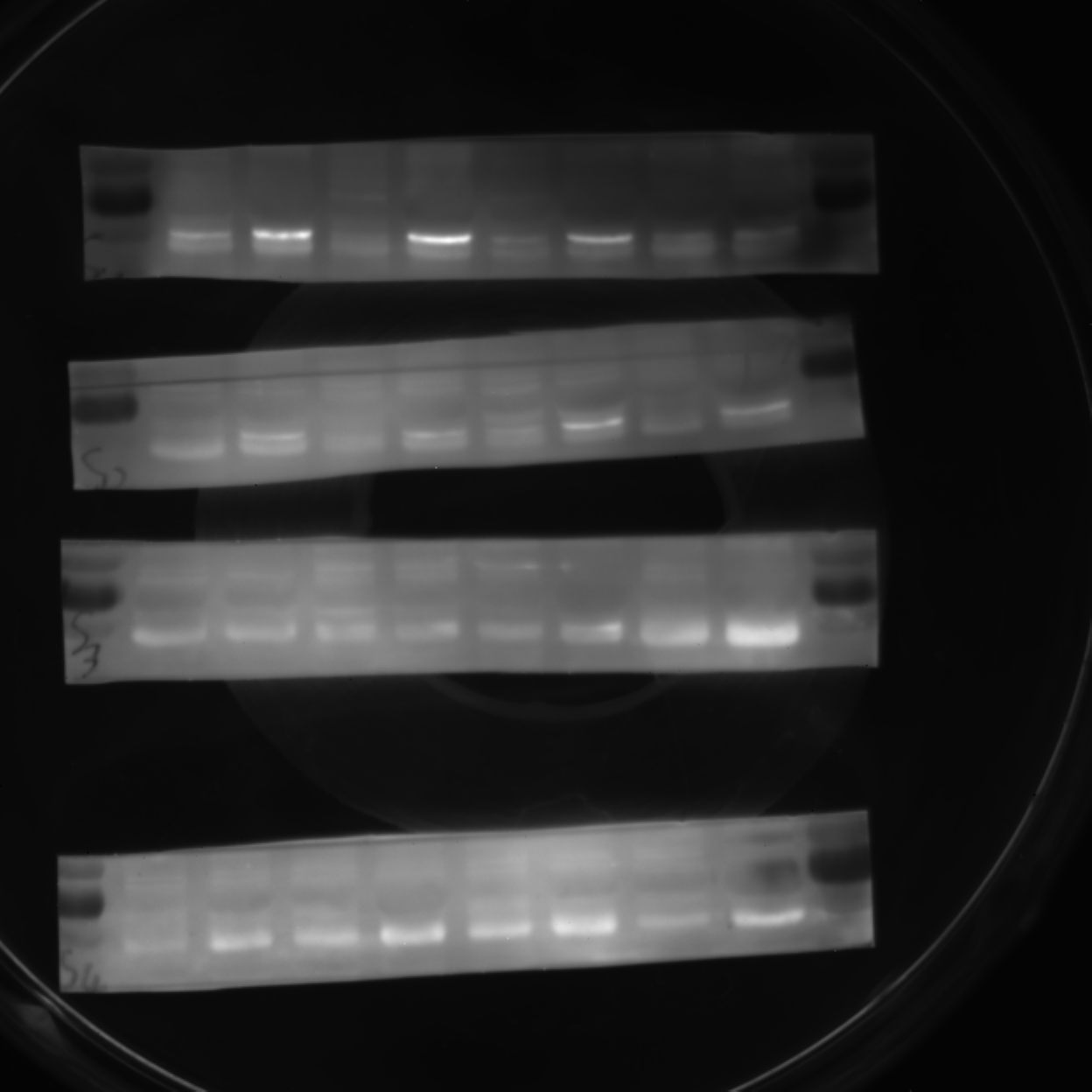

Supplement: S1 Fig — The image shows the blots of 9–24 paired samples, the first sample is cancer, the second one is the paired normal lung tissue, and so on. The first stripe is one in Fig 5C. (TIFF) [file pone.0203155.s011.tiff]

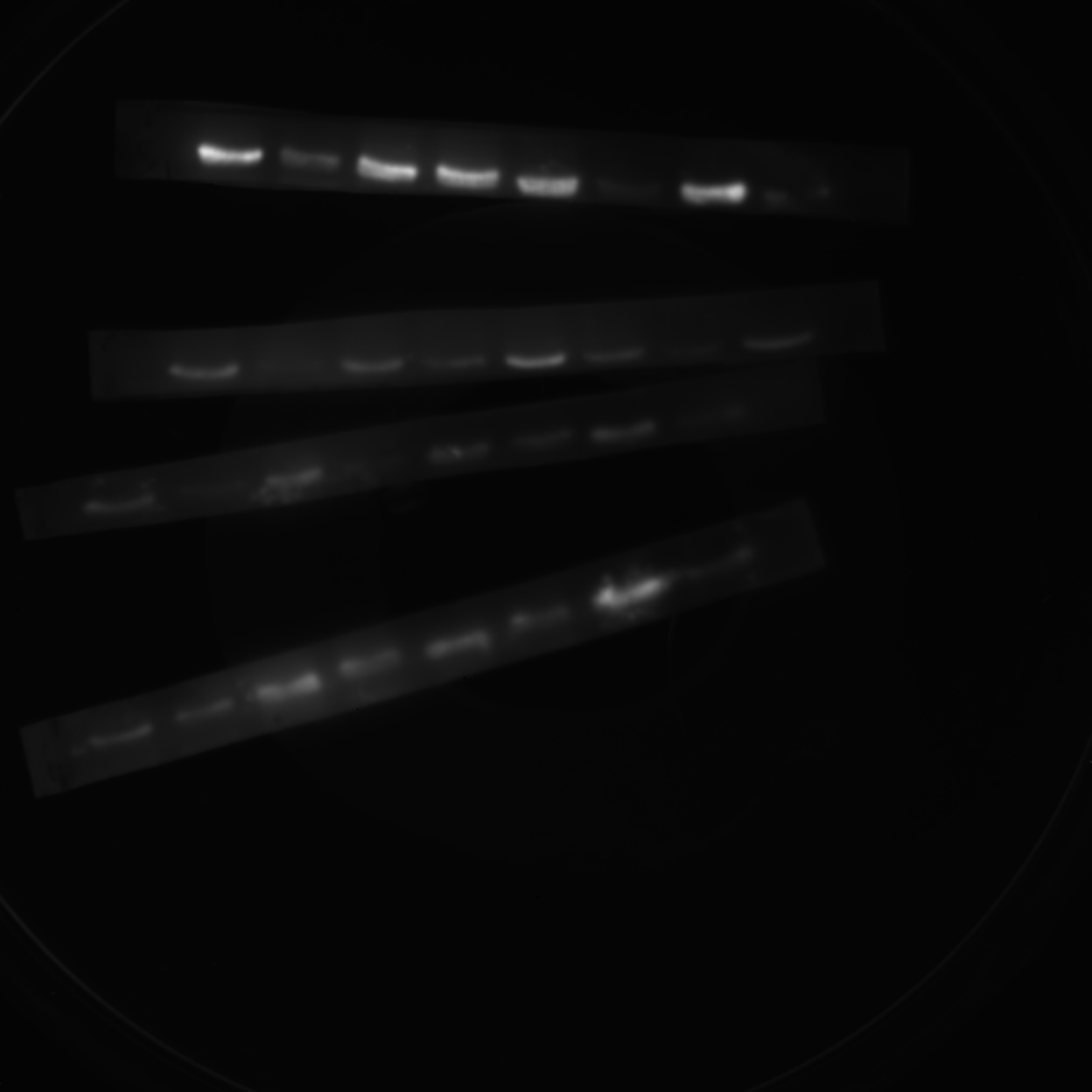

Supplement: S2 Fig — The image shows the blots of 9–24 paired samples, the first sample is cancer, the second one is the paired normal lung tissue, and so on. The first stripe is one in Fig 5C and related to S1 Fig. (TIFF) [file pone.0203155.s012.tiff]

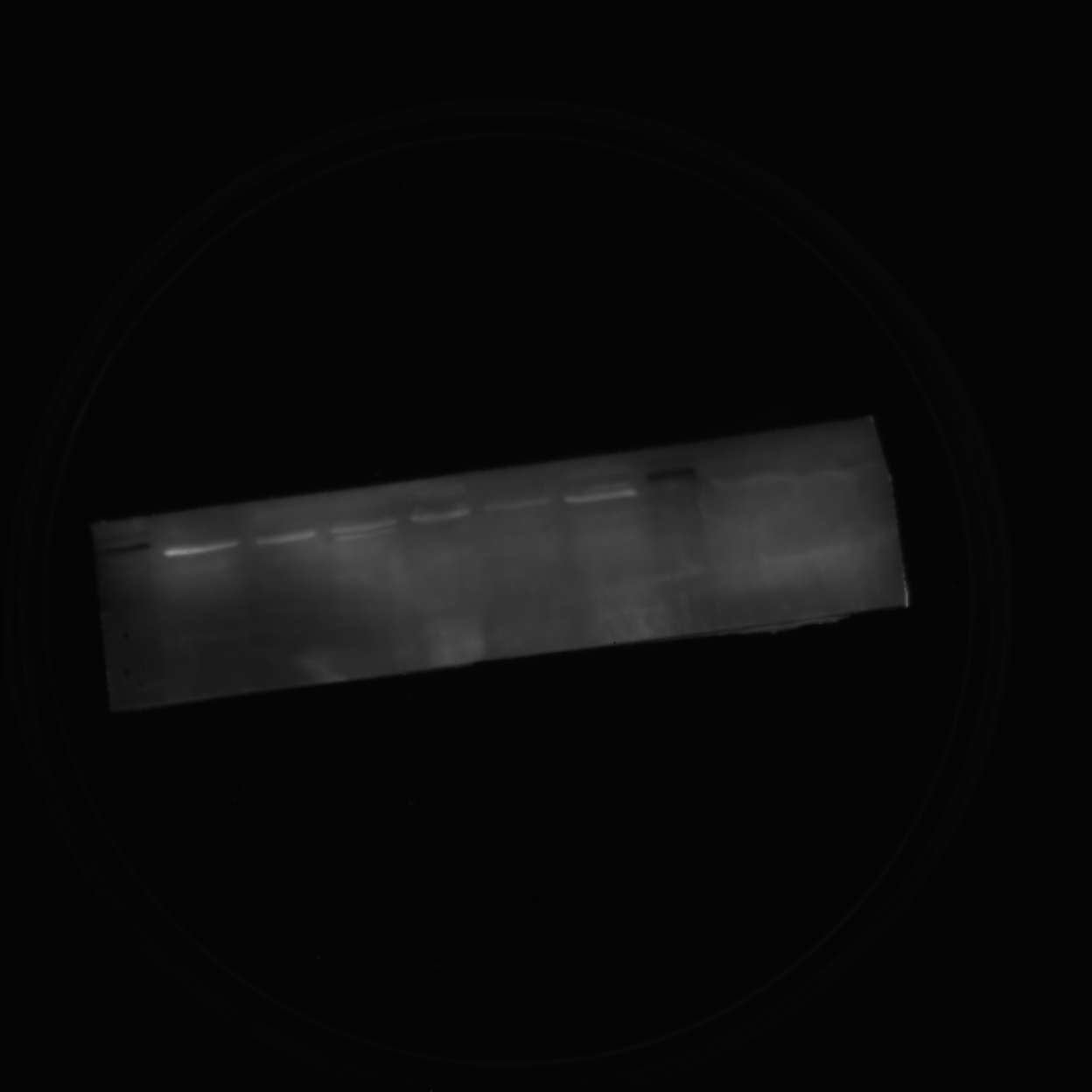

Supplement: S3 Fig — (TIFF) [file pone.0203155.s013.tiff]

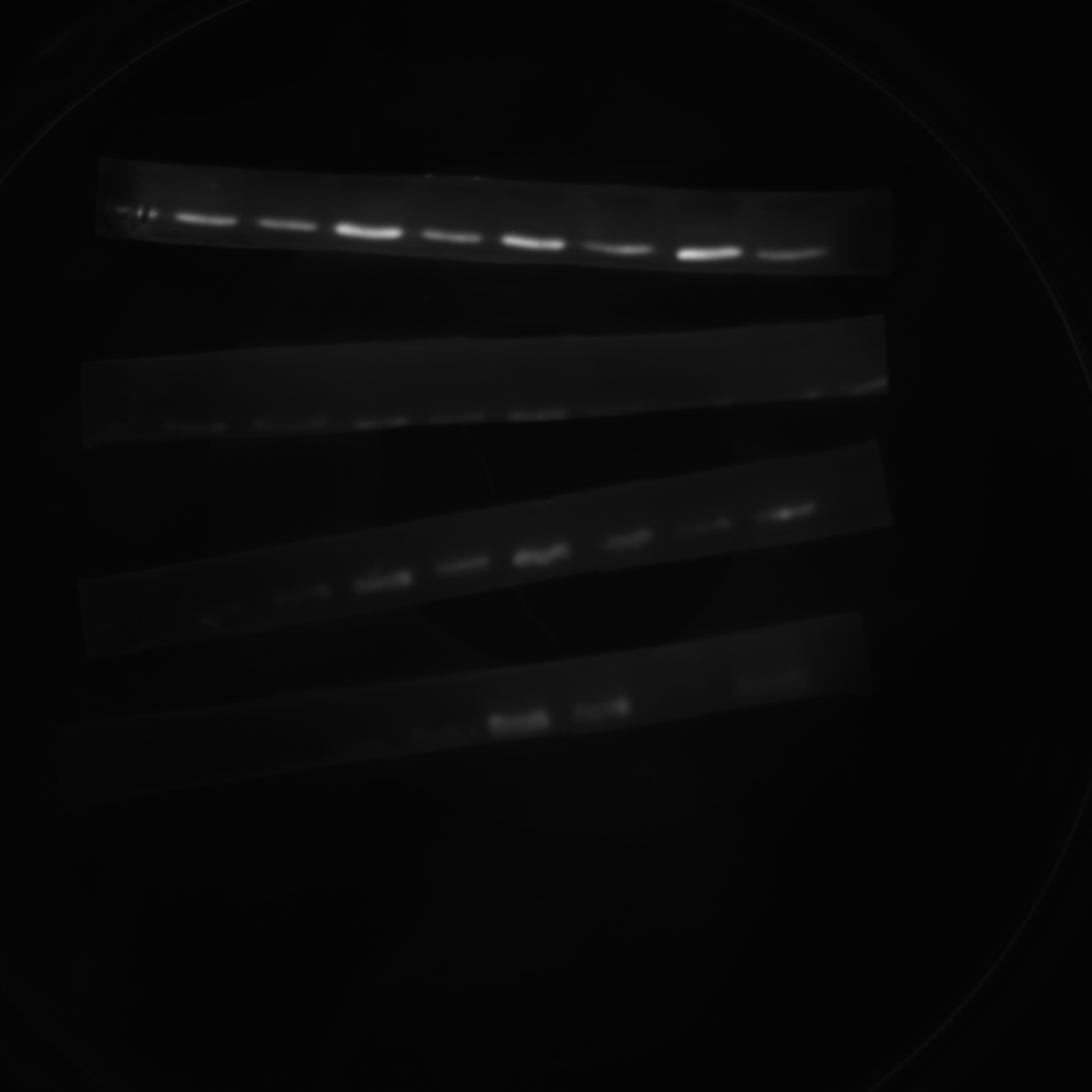

Supplement: S4 Fig — The image shows the blots of 9–24 paired samples, the first sample is cancer, the second one is the paired normal lung tissue, and so on. The first stripe is one in Fig 5C and related to S3 Fig. (TIFF) [file pone.0203155.s014.tiff]

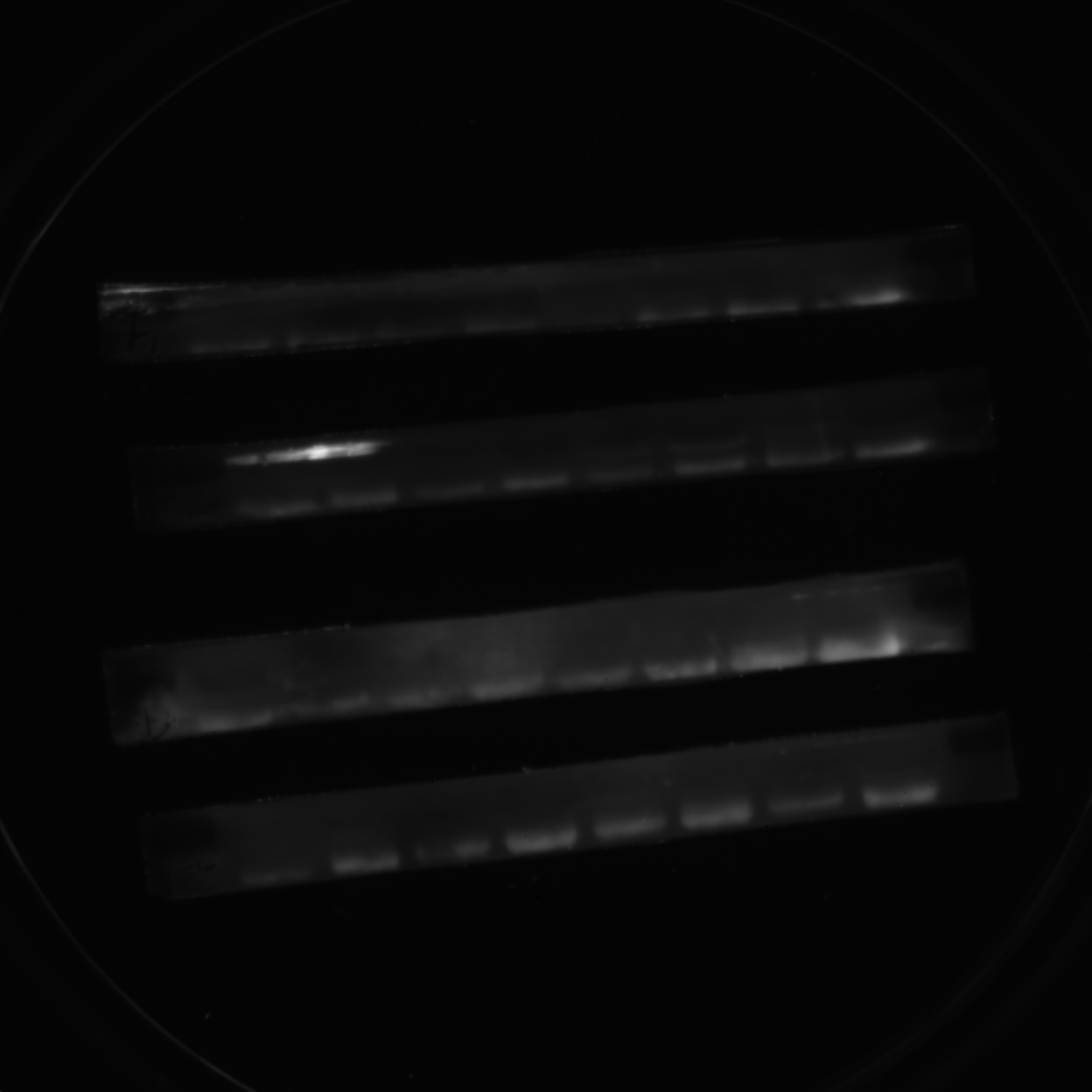

Supplement: S5 Fig — The image shows the blots of 9–24 paired samples, the first sample is cancer, the second one is the paired normal lung tissue, and so on. The fourth stripe is one in Fig 5C. (TIFF) [file pone.0203155.s015.tiff]

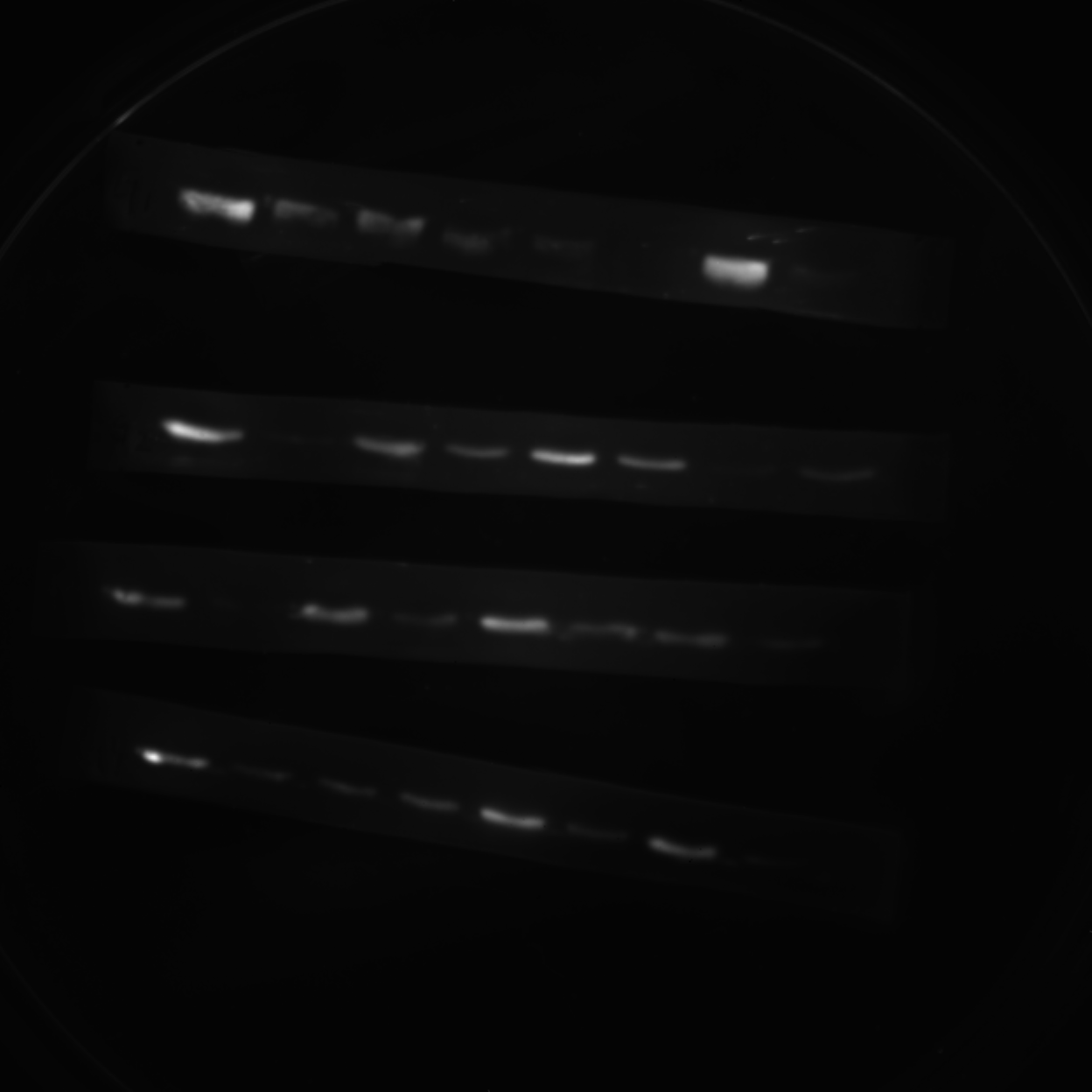

Supplement: S6 Fig — The image shows the blots of 9–24 paired samples, the first sample is cancer, the second one is the paired normal lung tissue, and so on. The fourth stripe is one in Fig 5C and related to S5 Fig. (TIFF) [file pone.0203155.s016.tiff]

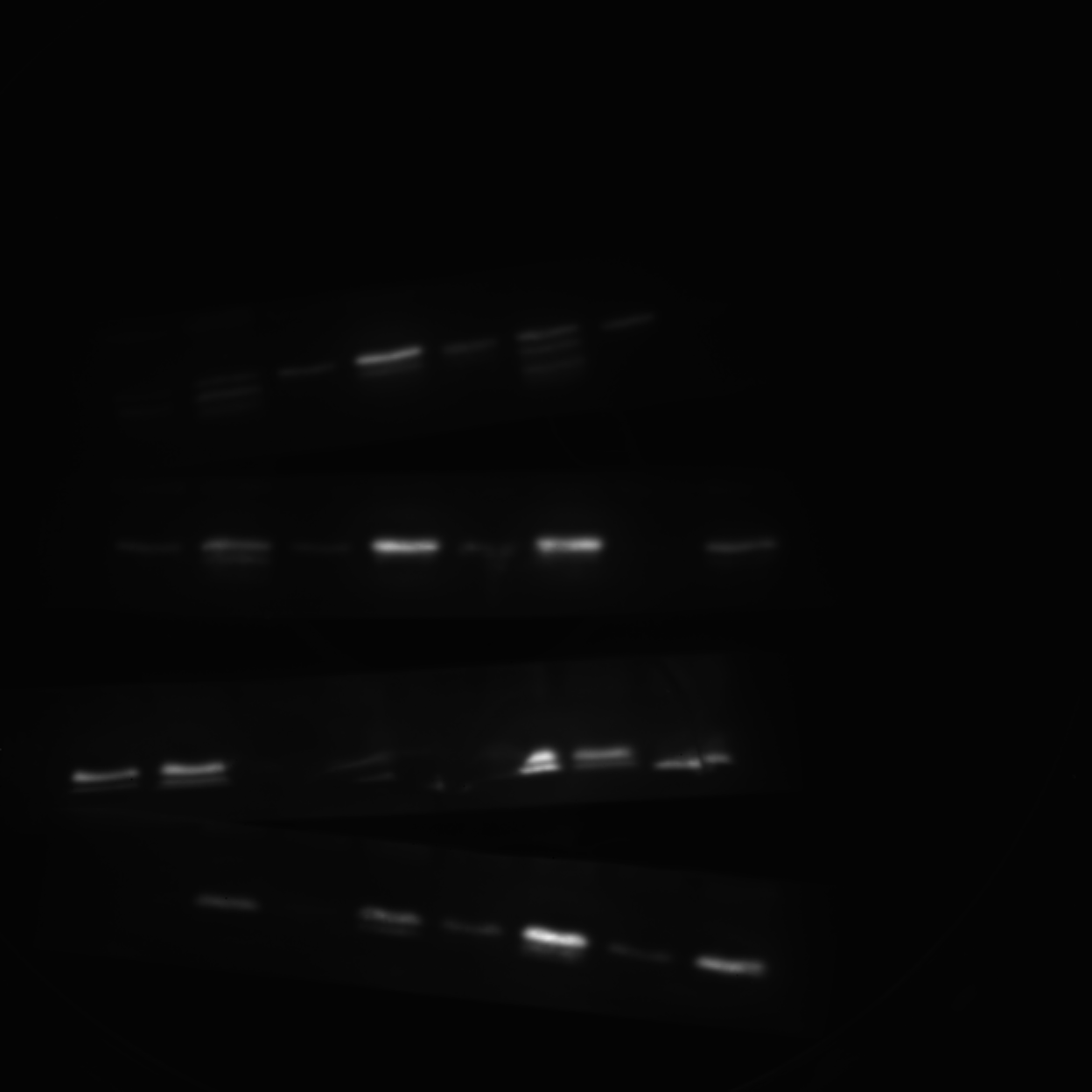

Supplement: S7 Fig — The image shows the blots of 9–24 paired samples, the first sample is cancer, the second one is the paired normal lung tissue, and so on. The second stripe is one in Fig 5C. (TIFF) [file pone.0203155.s017.tiff]

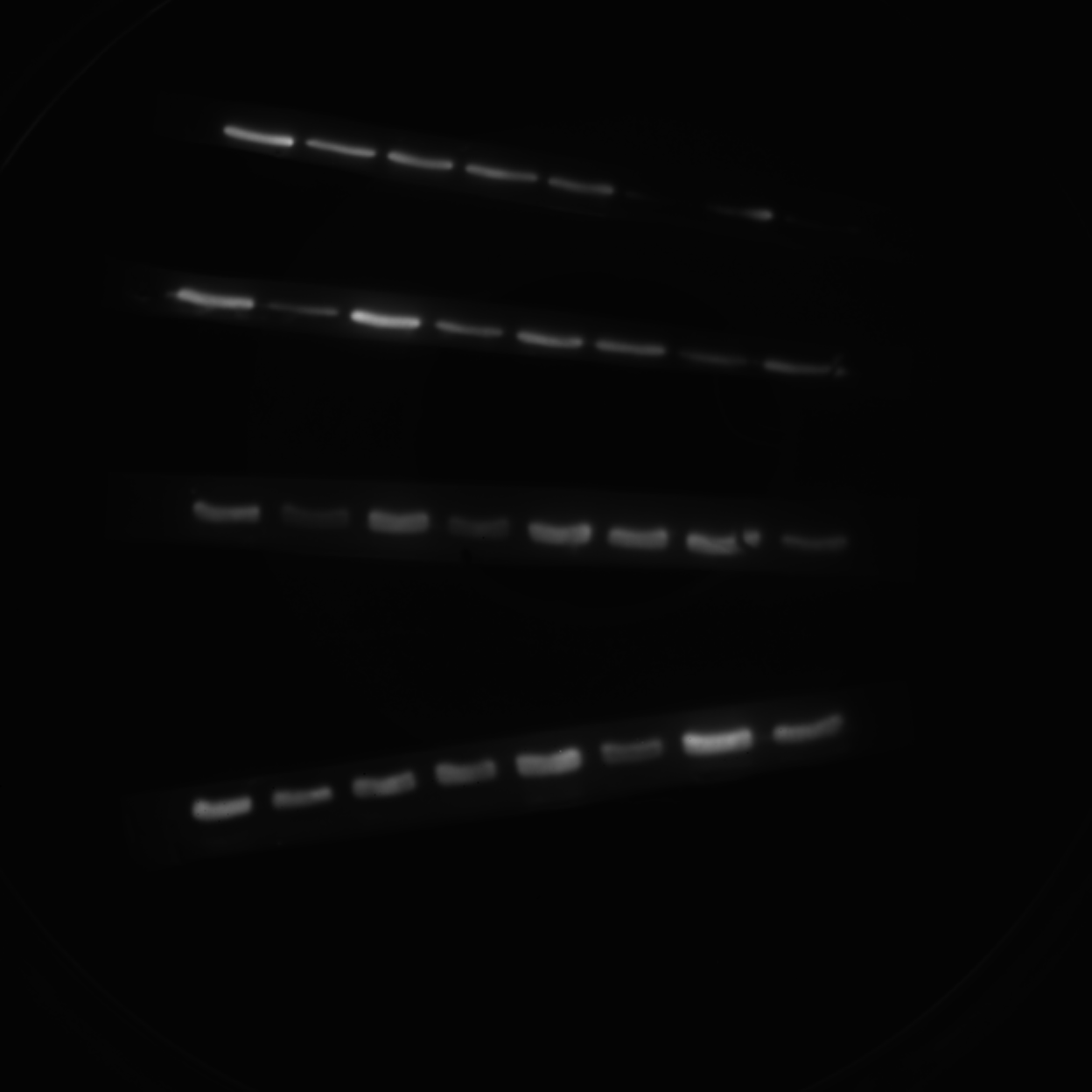

Supplement: S8 Fig — The image shows the blots of 9–24 paired samples, the first sample is cancer, the second one is the paired normal lung tissue, and so on. The second stripe is one in Fig 5C and related to S7 Fig. (TIFF) [file pone.0203155.s018.tiff]
